# The Water Footprint of Diets: A Global Systematic Review and Meta-analysis

**DOI:** 10.1093/advances/nmz091

**Published:** 2019-09-06

**Authors:** Francesca Harris, Cami Moss, Edward J M Joy, Ruth Quinn, Pauline F D Scheelbeek, Alan D Dangour, Rosemary Green

**Affiliations:** 1 Department of Population Health, London School of Hygiene and Tropical Medicine, London, United Kingdom; 2 Leverhulme Centre for Integrative Research on Agriculture and Health, London, United Kingdom; 3 Centre on Climate Change and Planetary Health, London School of Hygiene and Tropical Medicine, United Kingdom; 4 The School of Biological Sciences, University of Aberdeen, Aberdeen, United Kingdom

**Keywords:** food consumption, planetary health, sustainable diets, water use; environmental footprint

## Abstract

Agricultural water requirements differ between foods. Population-level dietary preferences are therefore a major determinant of agricultural water use. The “water footprint” (WF) represents the volume of water consumed in the production of food items, separated by water source; blue WF represents ground and surface water use, and green WF represents rain water use. We systematically searched for published studies using the WF to assess the water use of diets. We used the available evidence to quantify the WF of diets in different countries, and grouped diets in patterns according to study definition. “Average” patterns equated to those currently consumed, whereas “healthy” patterns included those recommended in national dietary guidelines. We searched 7 online databases and identified 41 eligible studies that reported the dietary green WF, blue WF, or total WF (green plus blue) (1964 estimates for 176 countries). The available evidence suggests that, on average, European (170 estimates) and Oceanian (18 estimates) dietary patterns have the highest green WFs (median per capita: 2999 L/d and 2924 L/d, respectively), whereas Asian dietary patterns (98 estimates) have the highest blue WFs (median: 382 L/d per capita). Foods of animal origin are major contributors to the green WFs of diets, whereas cereals, fruits, nuts, and oils are major contributors to the blue WF of diets. “Healthy” dietary patterns (425 estimates) had green WFs that were 5.9% (95% CI: −7.7, −4.0) lower than those of “average” dietary patterns, but they did not differ in their blue WFs. Our review suggests that changes toward healthier diets could reduce total water use of agriculture, but would not affect blue water use. Rapid dietary change and increasing water security concerns underscore the need for a better understanding of the amount and type of water used in food production to make informed policy decisions.

## Introduction

Food security depends on the availability of freshwater resources for agricultural production. Globally, ∼70% of freshwater is used annually for agricultural (food and nonfood) production. Climate change is projected to alter rainfall patterns and increase the occurrence of extreme weather events including more frequent droughts and floods (1). A growing human population and rapidly changing diets, including greater consumption of animal source foods (ASFs), has resulted in increasing global water use in agriculture (2). Identifying sustainable diets that promote health and minimize environmental impacts is increasingly important, and in this context, understanding the impact of food production and population-level dietary patterns on water use is critical for sustainable water management.

A growing body of literature suggests that in general a reduction in ASFs in the diet, particularly beef, poultry, and pork meat, corresponds with reduced environmental impacts and resource requirements (3–6). However, reducing ASF content of diets does not always correspond with lower water use, especially if ASF items are replaced with foods such as fruits and pulses that can be more dependent on irrigation (7). Additionally, there is large variability globally in the amount and type of water used in food production due to environmental and agricultural management factors (8). The most commonly used metric for assessing water use is the “water footprint” (WF), which quantifies the volume of water consumed during the production of an item (in liters per kilogram) and can be separated into the blue WF (representing the use of groundwater and surface water) and the green WF (representing the use of rainfall) (9). Crop WFs are primarily driven by evapotranspiration occurring in the field in which the crop is grown, whereas the WF of ASFs includes the evapotranspiration of feed crops and grazing lands as well as the animals’ drinking and service water needs. A high blue WF means that large volumes of irrigation water are used during crop production. This can be a concern in areas where surface water and groundwater reserves are being unsustainably exploited (10). A high green or total (green + blue) WF can indicate that crops have low yields or are inefficient in their water use. A low green and high blue WF suggests rainwater is being inefficiently used, which can lead to surface water and groundwater overexploitation. A previous systematic review assessed the water use of dietary patterns, but did not distinguish between green and blue water use nor did it consider spatial heterogeneity in WFs (5).

The aim of this systematic review was to collate and synthesize the available data on the global water use of human diets. First, we identified the available literature assessing the relation between diets and water use through the WF, outlining the different data sources and models used. Second, we explored heterogeneity in dietary WFs across the world, considering both blue and green water use. Finally, we identified the food groups that are most important in determining dietary WF, and using data from identified studies, we estimated the WFs of different dietary patterns.

## Methods

### Study selection and search strategy

We conducted this systematic review in accordance with the Preferred Reporting Items for Systematic Reviews and Meta-Analyses (PRISMA) guidelines (11). Included studies assessed human (population) diets (intervention) and their WF (outcome), published in English from 2000 up to the date of the search (7 February 2018) (including dietary WF estimates from 1995 onwards). We searched 7 online databases covering the fields of environment, social science, public health, nutrition, and agriculture: Web of Science Core Collection, Scopus, OvidSP MEDLINE, EconLit, OvidSP AGRIS, EBSCO GreenFILE, and OvidSP CAB Abstracts. References of previous reviews (5, 6) were hand-searched for additional articles.

The search was conducted with predefined search terms that included the concepts “diets” and “water footprint” (see **Supplemental Table 1** for all database-specific searches). After duplicates were removed, potentially relevant studies were assessed for inclusion by 2 independent researchers (FH, CM), and discrepancies were discussed and agreed by consensus. Eligible study designs included observational and modeling studies that quantified WFs from the perspective of dietary intake or food availability (known as the “bottom-up approach” in WF accounting) (12). Hence, we included studies that quantified diets through dietary intake surveys, food consumption and expenditure surveys, modeled dietary scenarios, and national food supply or availability accounts (amount of food available from production and imports after loss, exports, and other uses). Studies that only quantified future or projected dietary WFs were excluded.

### Data extraction and quality assessment

Data extraction from eligible studies included reference information, study setting, data sources of diets and water use, modeling assumptions used to link diets and WF, WF of the diet(s) with units (green, blue, and total), information on dietary pattern(s), and the top 2 food groups or food items contributing to the dietary WF. Most studies provided multiple dietary WF estimates, for example, for different dietary patterns, countries, or timescales. Only dietary WF estimates from recent past (since 1995) or current diets were extracted. If exact dietary WFs were not available through the published article or supplementary files but were presented graphically, precise estimates were requested from the study's corresponding author. We wrote to 13 authors, of whom 7 responded and sent additional data. To estimate the contribution of food groups in the diet, percentages were calculated where possible for inclusion in analysis.

The majority of studies included in the review were modeling studies (i.e., combining data from primary or secondary data sources), so we appraised study quality following an adjusted appraisal tool based on the Critical Appraisal Skills Programme (CASP) Randomized Controlled Trials Checklist (13) and the Questionnaire to Assess Relevance and Credibility of Modelling Studies (14). Our adjusted appraisal tool included 10 criteria, with studies scoring either “0” for not fulfilled, “1” for fulfilled, or “NA” if not applicable. Each study included in the review was graded based on its score and converted to a percentage, with <50% as low, 50–70% as medium, and >70% as high. This information was used to perform a sensitivity analysis removing studies of low quality. Data extraction and quality assessment were carried out by 2 independent researchers (FH, CM), and discrepancies resolved by consensus.

### Analysis

We tabulated information on the following features of included studies: location of study, scale (global, multicountry, national, subnational), WF assessed (green, blue, or total), data source for diets and WFs, and model assumptions used to link diet and water data. Green, blue, and total dietary WFs were standardized (to liters per day per capita). Subnational, national, and regional dietary WF estimates were categorized by continent and summary statistics calculated. National mean green, blue, and total WFs were calculated from national and subnational diet WF estimates and mapped using ArcGIS Desktop (Version 10.5; Environmental Systems Research Institute, Inc). Values were separated into 5 categories using Jenks optimization, defined by minimizing the within-category deviation from the mean, and maximizing the between-category deviation (15).

We explored the contribution of different food items to dietary WFs of each dietary pattern. Due to heterogeneity in study reporting and food groups assessed, we could not carry out a meta-analysis to explore the contribution of different food groups to the dietary WF. For example, studies reported food intake or availability in weighed amount (i.e., grams per day) or equivalent calories (i.e., kilocalories per day) and therefore could not be grouped. Additionally, some studies reported intake or availability based on specific food items (e.g., eggs or beef), whereas others reported in broad categories (e.g., ASFs). Therefore, we presented the top 2 contributing food groups or items to the dietary WF as stated in included studies. If available, percentage contributions were calculated, and, when multiple dietary WFs were estimated by the study, we recorded the range.

To assess the effect of diet pattern on the WF, we adopted a 1-step individual observation meta-analysis method using dietary WF estimates and diet pattern (16). Studies were only included in the meta-analysis if they provided an exact estimate of dietary WF that could be standardized to liters per day per capita. Meta-analysis was carried out using mixed effects regression models with study identifier as a random effect to account for multiple estimates from the same study. Dietary WFs were not normally distributed and therefore all regression analyses were carried out using log-transformed values.

Dietary patterns evaluated in included papers were grouped into 4 major categories as follows (**Supplemental Table 2** gives full details of categorization):

1. “Average” dietary patterns were those identified as current, baseline, or average intake in the included study. This category was used as the reference diet in statistical analysis.
2. “Healthy” dietary patterns were identified as such in the included study, therefore providing additional nutritional benefits when compared with average diets. These were typically national dietary guidelines [e.g., German Nutrition Society (17) or US Department of Agriculture (18)], or other food or nutrient-based guidelines [e.g., WHO (19)].
3. “Reduced animal source foods” included dietary patterns with lower consumption of ASFs than the average [e.g., those identified as vegetarian, or with step decreases in ASF content (e.g., −10%, −25%, etc.)].
4. “No animal source foods” meant no animal products consumed (e.g., those identified as vegan).

A few studies (*n* = 5) reported “other” dietary patterns, which included a small set of highly heterogeneous patterns including diets consumed by tourists and scenario diets that minimized WFs. These estimates were excluded from the meta-analysis.

Several models were used to quantify differences in dietary water use of each dietary pattern compared with the “average” dietary pattern. The WF values were log-transformed and regression coefficients were exponentiated, giving the proportional difference in dietary pattern relative to the average. The baseline model included dietary pattern, WF, and study identifier as a random effect. The location-adjusted model also included study location as a covariate. The fully adjusted model also adjusted for study scale, source of diet data, and source of WF data. Sensitivity analysis was performed by rerunning the analysis excluding studies graded as low quality (*n* = 2) and excluding studies contributing a large number of estimates (>500) (*n* = 2). It was not possible to test for publication bias, because SEs for the differences between the WFs of dietary patterns were not provided. All statistical analyses were conducted using STATA (v.15; StataCorp LP).

## Results

**FIGURE 1:**
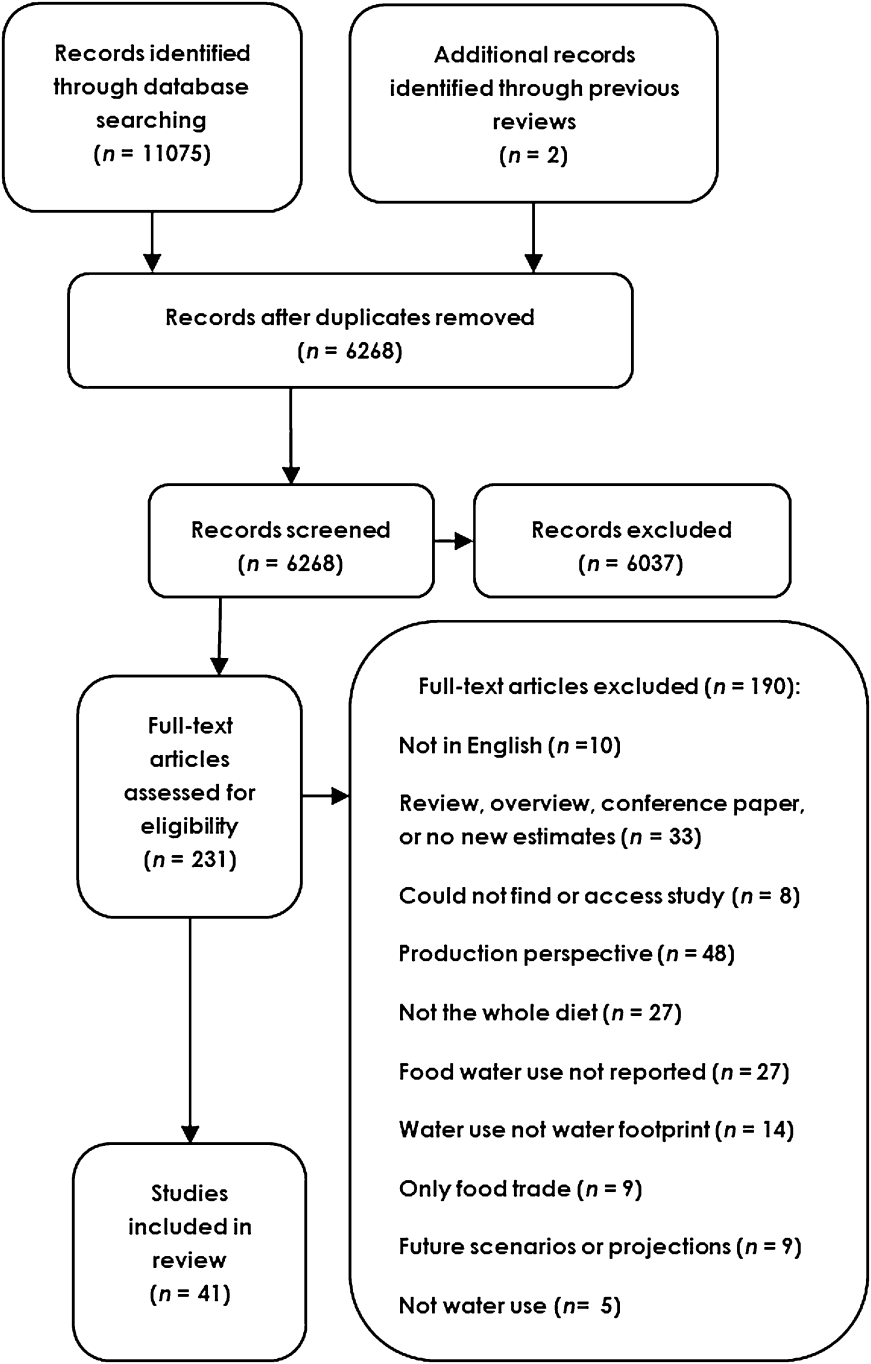
Flowchart, indicating identification and selection of studies.

Of 6268 unique studies identified in the initial search, a total of 41 studies were identified as relevant and included in this review (**Figure 1**). An additional 14 studies assessed dietary water use through metrics other than the WF, and were not included in this review (**Supplemental Table 3**).

### Study methods, context, and quality

**FIGURE 2:**
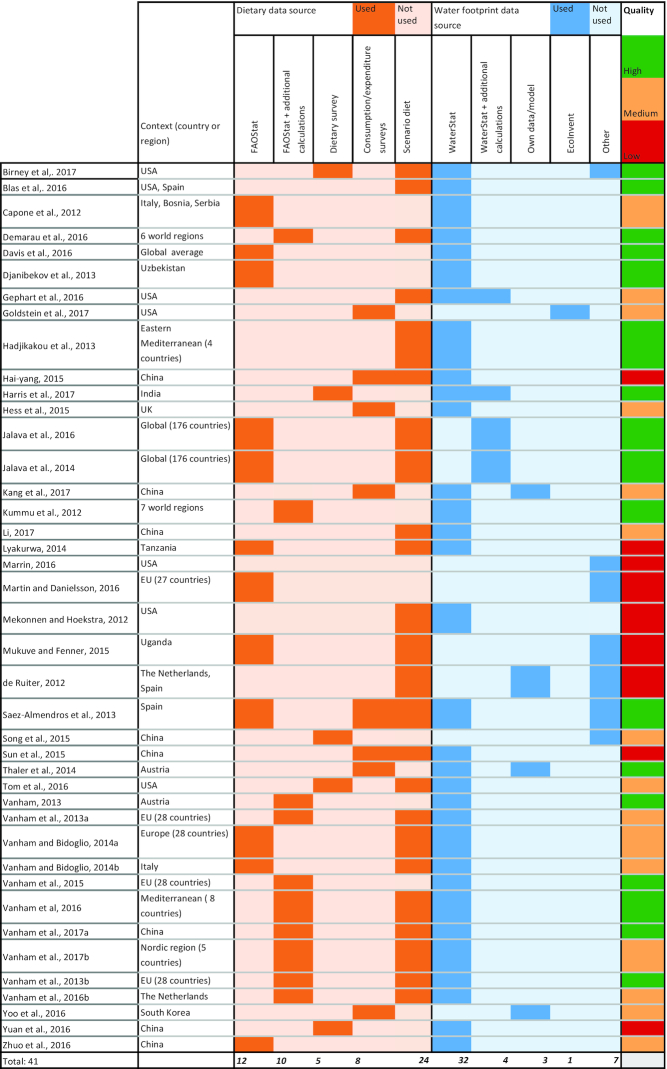
Characteristics of included studies: context, dietary and water use data, and quality (*n* studies = 41).

The included studies used a variety of data sources and methods (**Figure 2**; full details of each study are provided in **Supplemental Table 4**). Current dietary patterns were analyzed in 32 studies, and 66% (*n* = 21) of these used data on national food availability from UN FAO food balance sheets (FBSs) to derive dietary patterns. Most studies (*n* = 36) obtained WF data from the WaterStat database. Over half of the studies (*n* = 23) assessed dietary WFs at the national level. China (*n* = 8) (20–27) and the United States (*n* = 7) (28–34) had the highest number of subnational studies. A total of 17 studies assessed dietary WFs in Europe, either at regional (35–39), national (28,40–45), or subnational levels (46–50). Only 4 studies reported WFs of diets in low- or middle-income countries, namely Uzbekistan, India, Tanzania, and Uganda (51–54). One study quantified the dietary WFs for South Korea (55). Two large studies estimated national-level dietary WFs globally (176 countries) (56, 57). Three studies quantified regional or global average dietary WFs (58–60). A third of the studies assumed food was produced and consumed in the same area, and therefore the WFs of crop and livestock items were taken from that area (*n* = 16). Five studies accounted for food imports in their estimates of dietary WFs, but applied a global average WF value to imported items. Only 4 studies included models of food trade with weighted WFs based on countries of origin (20,28,40,41).

Of the 41 studies, 17 (41%) were graded as high quality and 9 (22%) as low quality (Figure 2,**Supplemental Table 5**). Only 4 studies provided a measure of uncertainty or variance for dietary WF estimates (24, 34, 52, 59). The quality of estimates included in regression analysis was high because the majority of estimates came from 3 high-quality studies (37,56, 57).

### Geographical variability in the WFs of current diets

**FIGURE 3:**
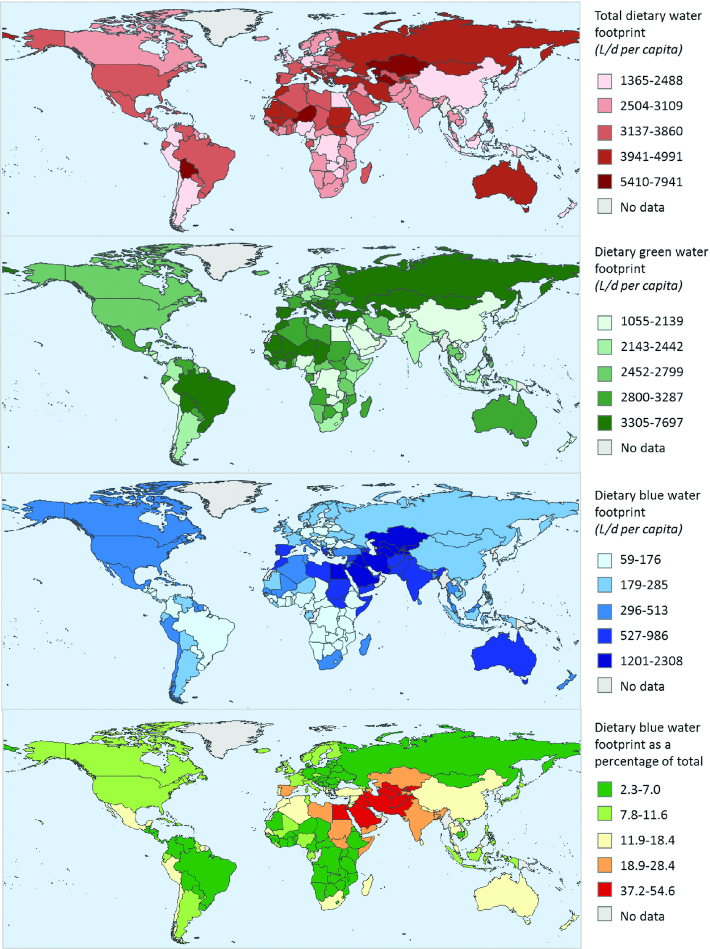
National dietary total, green, and blue dietary WFs, and blue WFs as a percentage of the total WF. Values are the mean for the respective country including national and subnational estimates. Categories are defined by natural breaks (15). WF, water footprint.

The WF of “average” dietary patterns varied depending on country and region (**Table 1**, **Figure 3**). Regionally, the total and green dietary WFs of “average” dietary patterns were greatest in Europe and Oceania. North American and Asian dietary patterns had the lowest total and green WFs. African diets had the lowest per capita median dietary blue WF, of 163 L/d (IQR: 118–267 L/d), whereas the WFs of dietary patterns in Asia were nearly double this at 382 L/d (IQR: 239–663 L/d). “Average” dietary patterns in Asia also had the greatest blue WF as a percentage of total dietary WF. “Average” dietary patterns in Egypt and Uzbekistan were more dependent on blue than green water, with blue WF representing 54% and 52% of total WF, respectively. In all other countries, “average” dietary patterns were more dependent on green than blue water. The lowest dependency on blue water was in Chad and Eritrea, where only 2% of the total dietary WF was blue.

**TABLE 1:** Summary of green, blue, and total WFs of the “average” dietary patterns in each continent^1^

	Green^2^ WF		Blue^3^ WF		Total^4^ WF	
Continent	Median (IQR), L/d per capita	*n* Estimates	Median (IQR), L/d per capita	*n* Estimates	Median (IQR), L/d per capita	*n* Estimates
Africa	2681 (2324–3159)	97	163 (118–267)	98	2846 (2489–3471)	98
Asia	2321 (1762–2779)	96	382 (239–663)	98	2862 (2238–3541)	100
Europe	2999 (2604–3642)	152	241 (159–366)	153	3227 (2873–3792)	170
North America	2370 (2108–2949)	51	220 (144–300)	54	2617 (2252–3214)	51
Oceania	2924 (2361–3402)	18	230 (220–322)	18	3226 (2579–3632)	18
South America	2735 (2013–3574)	25	202 (152–296)	26	2932 (2322–3730)	25

1 WF, water footprint.

2 Volume of rainfall water consumed in the production of the diet.

3 Volume of ground and surface water consumed in the production of the diet.

4 Green and blue WFs combined.

### Major foods contributing to the dietary WF

Data on the contribution of foods to WF were available in 30 studies (**Supplemental Table 6**). Here, food and food groups refer to both commodities as defined in FAO FBSs, as well as food ready for human consumption (see Supplemental Table 6). ASFs, particularly meats, were the major component of total and green dietary WFs of “average” dietary patterns. Cereals were the second most important foods for total and green dietary WFs. Plant-based foods, including cereals, nuts, and sugar, were the major components of blue WFs of “average” dietary patterns, although ASFs were still in the top 2 contributing foods in 5 out of the 10 studies. Switching to healthier diets changes the contribution of foods to the dietary WF. Plant-based foods feature as major contributors to total and green dietary WFs in 6 of 8 studies. Plant-based foods still dominated the blue WFs of healthy diets, with the inclusion of fruits as a major contributor.

The contribution of food groups to dietary WFs in “reduced ASF” or “no ASF” dietary patterns was only reported in 8 studies. In “reduced ASF” patterns, meat was usually reduced first before other animal products. Therefore, in the “reduced ASF” dietary patterns, the contribution to dietary WF of items such as milk increases relative to that of meat. Additionally, products such as tea and coffee become major contributors to the total dietary WF. Only 1 study reported the contribution of food to the dietary WF for the “no ASF” pattern; fruits and vegetables accounted for 34% of the dietary blue WF of this pattern in the United States (31).

### Meta-analysis of dietary patterns and water use

**FIGURE 4:**
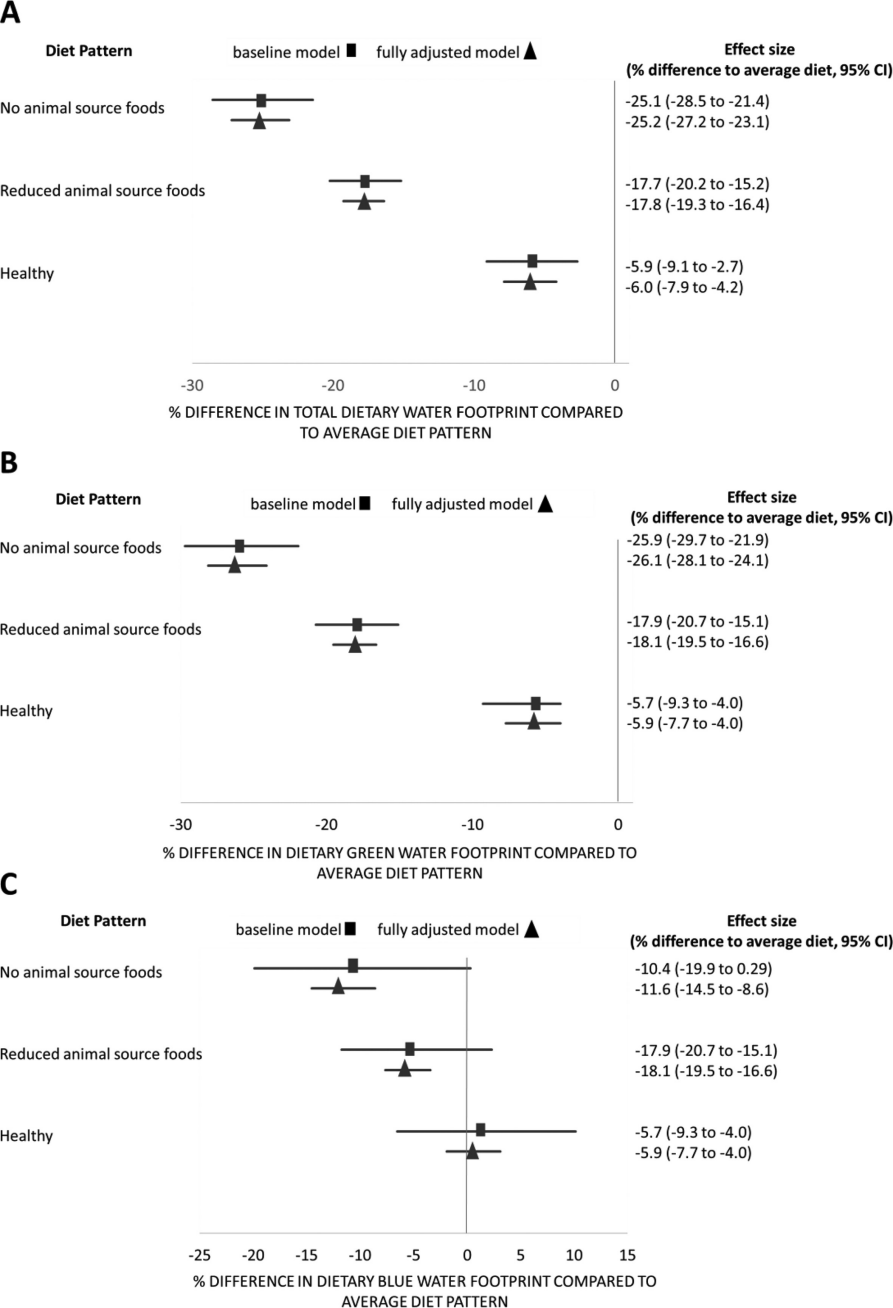
Forest plots with coefficient estimates from the mixed effects regression of diet pattern and (A) total WF, (B) green WF, and (C) blue WF. Values represent the percentage differences (95% CI) in dietary WF for each of the 3 dietary patterns compared with the average dietary pattern; *n* studies = 32 (total), 20 (green), and 24 (blue); *n* estimates = 1933 (total), 1834 (green), and 1895 (blue). In all graphs the 0 line represents the “average” dietary pattern. Study identifier was used as a random effect, and the fully adjusted model included study location, scale, and source of WF data. WF, water footprint.

In total, 1964 individual dietary WF estimates from 36 studies were available for inclusion in the meta-analysis to determine the WF of different dietary patterns (**Figure 4**, **Supplemental Tables 7–9**). Five studies reporting 28 estimates were excluded from the meta-analysis, because it was not possible to convert reported dietary WF estimates to liters per day per capita (22, 32,33,35, 54). Compared with “average” dietary patterns, “healthy” dietary patterns, “reduced ASF” dietary patterns, and “no ASF” dietary patterns had significantly lower total and green WFs (Figure 4). Adjusting for study location and other characteristics improved the precision of the models, suggesting there is some variability in the size of relation depending on study context. The WF of the “no ASF” pattern differed most markedly from the “average” pattern, with the total WF 25.2% lower after adjusting (95% CI: −27.1, −23.1; *P* < 0.001) and green WF 26.1% lower after adjusting (95% CI: −28.1, −24.1; *P* < 0.001). The healthier patterns had a slightly lower total WF (adjusted percentage difference: −6.0%; 95% CI: −7.9, −4.2; *P* < 0.001) and green WF (adjusted percentage difference: −5.9%; 95% CI: −7.7, −4.0; *P* < 0.001) than that of “average” dietary patterns. We found no evidence of a difference between the blue WF of “healthy” and “average” dietary patterns, even after adjusting for study location and characteristics. In the fully adjusted model there was evidence that “no ASF” and “reduced ASF” dietary patterns had lower blue WFs compared with the “average” dietary pattern (adjusted percentage difference: −11.6%; 95% CI: −14.5, −8.6; *P* < 0.001, and −5.6%; 95% CI: −7.6, 3.4; *P* < 0.001, respectively). However, this varied from the unadjusted model suggesting the relation is dependent on study location.

Findings from sensitivity analysis that excluded studies of low quality did not differ from the original analysis. Sensitivity analysis that excluded the 2 large studies that each provided >500 dietary WF estimates, reduced the robustness of the findings largely due to reduced data availability (Supplemental Tables 7–9).

## Discussion

### Summary of findings

This systematic review reports the available published evidence assessing the relation between human diets and the water used in their production. The average WF of diets ranged from 616 to 8075 L/d per capita for green water, 40 to 2450 L/d per capita for blue water, and 688 to 8341 L/d per capita for the total water use. Our review identified large geographical differences in the water use of diets: green WFs of diets were greatest in Europe, whereas blue WFs of diets were greatest in Asia. ASFs were major contributors to green and total WFs, whereas plant-based foods were more dominant in the dietary blue WFs. Our new analysis, including data from 36 studies, suggests that switching from current “average”’ dietary patterns to “healthier” diets would result in decreased green WFs, but might not reduce blue WFs. Compared with “average” dietary patterns, reducing the ASF content of diets would reduce green WFs, and in most cases blue WFs.

### Research in context

To our knowledge, this is the first global systematic review of the WFs of diets. We included 41 relevant articles that reported 1964 WF estimates from 176 countries and were able to compare the blue, green, and total WFs of different dietary patterns. By combining estimates from multiple studies, we assessed the spatial variability in dietary WFs and provided summary estimates by continent. Considerable heterogeneity exists in the total water use of diets and in the relative proportions of green and blue WFs to total WFs. Some of this variation can be attributed to local climate and agricultural management factors. For example, dietary blue WFs were much greater in areas such as the Middle East where there is limited rainfall and a greater need for irrigation. Additionally, our review highlighted that differences in composition of the diet could explain some of this variation. ASFs were the main contributor to the total WF of diets, and total dietary WF was greater in areas with high ASF consumption, such as Europe and Oceania, compared with the global average (61).

Concurrently, our study demonstrated that switching to diets with “no ASF” from current “average” dietary patterns would decrease total WF by 25% and blue WF by 12%. The total WFs of “reduced ASF” dietary patterns were also lower than “average” patterns. Dairy products typically have a lower WF than meat (33), and the reduced ASF patterns often substituted the meat with dairy products, oil crops, and pulses. One previous review that assessed dietary WFs in 8 mostly high-income countries, also reported that vegetarian diets had lower total WFs compared with current habits, and that changing to healthier dietary patterns would result in a median reduction in total dietary WF of 18% (5). Our new analysis includes data from 176 countries and is therefore more representative of global food systems. Our estimate of the potential for healthier dietary patterns to reduce total WF was lower (−6.0%; 95% CI: −7.9, −4.2), perhaps reflecting the greater diversity in current “average” diets. For example, particularly in low-income settings, diets might need to increase their ASF content to achieve nutritional adequacy, thereby concomitantly increasing the dietary WF (61, 62). Our study shows that “healthier” diets have blue WFs similar to “current” dietary patterns. Plant-based foods that are important components of healthy diets, such as fruits, oils, and nuts, were major contributors to dietary blue WFs (63). Production of these crops, and therefore healthy diets, could be sensitive to declining groundwater or surface water availability where this might limit irrigation (64).

### Strengths and limitations

By pulling together the available evidence on dietary WFs, this review adds to the growing literature on the environmental impacts of human diets, and the potential for dietary change to reduce this impact. We systematically sought and reviewed the available evidence from 7 databases and identified significantly more studies than previous reviews (4, 5). We prespecified inclusion criteria, and 2 independent reviewers assessed each publication for relevance. We included studies that modeled diets, but did not include studies that assessed the WFs of diets projected into the future, due to the associated uncertainties of such projections (65, 66). Several indicators have been applied to assess the relation between diets and water use. The available evidence base is dominated by the WF and this review did not incorporate findings that used alternative metrics of water use.

Dietary WF assessments predominantly rely on 2 major open data sources [FAOSTAT FBSs (67) and WaterStat (33, 68)] that both have limitations. The FBSs report data on per capita food availability at the national level, and although these data are frequently used as a proxy for individual dietary intake, they typically overestimate actual dietary intake (69) and can therefore overestimate dietary WFs. Data from WaterStat are relatively outdated (1996–2005), and make use of globally gridded databases that might not adequately account for variation. For example, the database on ASFs relies on estimates aggregated to geoeconomic region such as Asia or member countries of the Organization for Economic Cooperation and Development (OECD) (33). To accurately estimate dietary WFs an understanding of where the food is produced and consumed is needed, and yet a third of studies (*n* = 16) did not incorporate any information on food trade in their models. Our analysis used location of where the diet would be consumed, rather than location of crop production, to estimate spatial variability in WFs, and we recognize that this will have underestimated the variability in WF. Furthermore, because the available published literature has mainly focused on high-income settings, there is limited representation of production systems in low- and middle-income countries, which might have different WFs. For example, the type of livestock system can affect both the type and amount of water used by the feed products, and therefore the associated WF of the ASF (70). These differences have not been explored here.

Poor reporting of methods, modeling approaches, and data sources were all common in the included studies, and there was a lack of uncertainty estimates. Sensitivity analysis removing studies of low quality did not lead to any differences in the interpretation of our regression results. The challenge of diverse reporting standards across academic disciplines and subsequent synthesis has been identified in previous interdisciplinary reviews (3). Finally, our meta-analysis was particularly dependent on 2 large studies from the same author group (56, 57), and as highlighted above, the majority of studies were focused on high-income settings. This identifies the need for more evidence on dietary WFs to be generated by academic groups around the world.

### Policy relevance and further research needs

By synthesizing the available literature, we provide estimates for the WFs of human diets for each continent. This is important for food security and environmental sustainability, because considerable spatial heterogeneity exists, which indicates both solutions and risks. For example, dietary blue WFs in Asia were found to be particularly high. Water scarcity in this region is a concern because groundwater resources are depleting in some areas, and climate change could disrupt normal patterns of rainfall and irrigation water supply (10, 71, 72). Changing dietary habits in Asia therefore could be insufficient to reduce local water use, unless coinciding with improved water management in agriculture (7, 73). Instead, improvements to nutritional status could be achieved through switching to more nutrient-dense and water-efficient crops. For example, it has been shown that cereals such as maize, millet, and sorghum could be grown instead of rice and wheat in India (74, 75). Countries could also import food from water-abundant regions.

Our findings also demonstrate that changes to current dietary patterns could be beneficial for both health and water sustainability. Healthy diets have a lower total WF compared with current patterns, and reducing ASFs could further decrease this. However, the evidence for blue WFs was not well defined. Fruit, nuts, and vegetables were major components of dietary blue WFs, particularly in healthy patterns. Literature on sustainable diets has generally focused on the importance of reducing ASFs to reduce environmental impacts (63). Future research needs to consider fruits, vegetables, and nuts in more detail, particularly because an increase in production is required to meet healthy dietary guidelines globally (76).

To understand the full impact of consumers on water resources, water use must be linked to local water availability, particularly in areas where water demand is growing and climate change threatens supply. Some studies are now using a water scarcity–weighted footprint metric for this purpose (41, 77), but such studies remain relatively rare. Additionally, food trade must be considered in future research, because it affects dietary WF calculations and could offer potential solutions to reduce local WFs in areas of water scarcity. Development of new technologies to record food supply chains will enable more accurate assessments of WFs in the future, and will help to inform policy and consumer decisions.

## Supplementary Material

nmz091_Supplemental_File

## Notes

This study forms part of the Sustainable and Healthy Food Systems (SHEFS) program supported by the Wellcome Trust's Our Planet, Our Health program (grant number: 205200/Z/16/Z).

Author disclosures: FH, CM, EJMJ, RQ, PFDS, ADD, and RG, no conflicts of interest.

Supplemental Tables 1–9 are available from the “Supplementary data” link in the online posting of the article and from the same link in the online table of contents at https://academic.oup.com/advances/.

Abbreviations used: ASF, animal source food; FBS, food balance sheet; WF, water footprint.

## References

[1] Schewe J, Heinke J, Gerten D, Haddeland I, Arnell NW, Clark DB, Dankers R, Eisner S, Fekete BM, Colón-González FJ Multimodel assessment of water scarcity under climate change. Proc Natl Acad Sci. 2014;111(9):3245–50. 24344289 10.1073/pnas.1222460110 PMC3948304

[2] Vörösmarty CJ, Green P, Salisbury J, Lammers RB Global water resources: vulnerability from climate change and population growth. Science. 2000;289(5477):284–8. 10894773 10.1126/science.289.5477.284

[3] Hallström E, Carlsson-Kanyama A, Börjesson P Environmental impact of dietary change: a systematic review. J Cleaner Prod. 2015;91:1–11.

[4] Jones AD, Hoey L, Blesh J, Miller L, Green A, Shapiro LF A systematic review of the measurement of sustainable diets. Adv Nutr. 2016;7(4):641–64. 27422501 10.3945/an.115.011015 PMC4942861

[5] Aleksandrowicz L, Green R, Joy EJ, Smith P, Haines A The impacts of dietary change on greenhouse gas emissions, land use, water use, and health: a systematic review. PLoS One. 2016;11(11):e0165797. 27812156 10.1371/journal.pone.0165797 PMC5094759

[6] Ridoutt BG, Hendrie GA, Noakes M Dietary strategies to reduce environmental impact: a critical review of the evidence base. Adv Nutr. 2017;8(6):933–46. 29141975 10.3945/an.117.016691 PMC5682998

[7] Springmann M, Clark M, Mason-D'Croz D, Wiebe K, Bodirsky BL, Lassaletta L, de Vries W, Vermeulen SJ, Herrero M, Carlson KM Options for keeping the food system within environmental limits. Nature. 2018;562(7728):519–25. 30305731 10.1038/s41586-018-0594-0

[8] Mekonnen MM, Hoekstra AY. Water footprint benchmarks for crop production: a first global assessment. Ecol Indic. 2014;46:214–23.

[9] Aldaya MM, Chapagain AK, Hoekstra AY, Mekonnen MM The water footprint assessment manual: setting the global standard. Routledge; 2012.

[10] Mekonnen MM, Hoekstra AY Four billion people facing severe water scarcity. Sci Adv. 2016;2(2):e1500323. 26933676 10.1126/sciadv.1500323 PMC4758739

[11] Moher D, Liberati A, Tetzlaff J, Altman DG; the PRISMA group. Preferred Reporting Items for Systematic Reviews and Meta-Analyses: the PRISMA statement. PLoS Med. 2009;6(7):e1000097. 19621072 10.1371/journal.pmed.1000097 PMC2707599

[12] Feng K, Chapagain A, Suh S, Pfister S, Hubacek K Comparison of bottom-up and top-down approaches to calculating the water footprints of nations. Econ Syst Res. 2011;23(4):371–85.

[13] CASP. CASP appraisal checklists. [Internet]. [cited October 10, 2017]. Available from: https://casp-uk.net/casp-tools-checklists/.

[14] Caro JJ, Eddy DM, Kan H, Kaltz C, Patel B, Eldessouki R, Briggs AH Questionnaire to assess relevance and credibility of modeling studies for informing health care decision making: an ISPOR-AMCP-NPC Good Practice Task Force report. Value Health. 2014;17(2):174–82. 24636375 10.1016/j.jval.2014.01.003

[15] Jenks GF. The data model concept in statistical mapping. In: International yearbook of cartography. 1967;7:186–90.

[16] Riley RD, Lambert PC, Abo-Zaid G Meta-analysis of individual participant data: rationale, conduct, and reporting. BMJ. 2010;340:c221. 20139215 10.1136/bmj.c221

[17] Elmadfa I European nutrition and health report 2009. Karger Medical and Scientific Publishers; 2009.

[18] United States Department of Agriculture. 2015–2020 dietary guidelines for Americans. 8th ed Washington DC: US Department of Health and Human Services; 2015.

[19] WHO. Diet, nutrition, and the prevention of chronic diseases: report of a joint WHO/FAO expert consultation: World Health Organization; 2003.

[20] Vanham D, Gawlik BM, Bidoglio G Cities as hotspots of indirect water consumption: the case study of Hong Kong. J Hydrol (Amst). 2019;573:1075–86. 31293281 10.1016/j.jhydrol.2017.12.004 PMC6588220

[21] Hai-yang S. The comparison of virtual water consumption among the various consumption patterns of diet. Adv J Food Sci Technol. 2015;7(11):875–80.

[22] Kang JF, Lin JY, Zhao XF, Zhao SN, Kou LM Decomposition of the urban water footprint of food consumption: a case study of Xiamen City. Sustainability. 2017;9(1):135.

[23] Li J. Scenario analysis of tourism's water footprint for China's Beijing-Tianjin-Hebei region in 2020: implications for water policy. J Sustain Tour. 2018;26(1):127–45.

[24] Song G, Li M, Semakula HM, Zhang S Food consumption and waste and the embedded carbon, water and ecological footprints of households in China. Sci Total Environ. 2015;529:191–7. 26011615 10.1016/j.scitotenv.2015.05.068

[25] Sun S, Wang Y, Wang F, Liu J, Luan X, Li X, Zhou T, Wu P Alleviating pressure on water resources: a new approach could be attempted. Sci Rep. 2015;5:14006. 26364756 10.1038/srep14006 PMC4650578

[26] Yuan Q, Song GB, Zhang SS Water footprint of household food consumption in Heilongjiang Province, China. In: 2016 International Conference on Sustainable Energy, Environment and Information Engineering (Seeie 2016); March 20–21, 2016, Bangkok, Thailand DEStech Publications, Inc; 2016:77–80.

[27] Zhuo L, Mekonnen MM, Hoekstra AY Water footprint and virtual water trade of China: past and future. Delft, the Netherlands: UNESCO-IHE Institute for Water Education. Value of Water Research Report Series No. 69, 2016.; 10.1016/j.envint.2016.05.019 27262784

[28] Blas A, Garrido A, Willaarts B Evaluating the water footprint of the Mediterranean and American diets. Water (Switzerland). 2016;8(10):448.

[29] Birney CI, Franklin KF, Davidson FT, Webber ME An assessment of individual foodprints attributed to diets and food waste in the United States. Environ Res Lett. 2017;12(10):105008.

[30] Gephart JA, Davis KF, Emery KA, Leach AM, Galloway JN, Pace ML The environmental cost of subsistence: optimizing diets to minimize footprints. Sci Total Environ. 2016;553:120–7. 26906699 10.1016/j.scitotenv.2016.02.050

[31] Goldstein B, Moses R, Sammons N, Birkved M Potential to curb the environmental burdens of American beef consumption using a novel plant-based beef substitute. PLoS One. 2017;12(12):e0189029. 29211775 10.1371/journal.pone.0189029 PMC5718603

[32] Marrin DL. Using water footprints to identify alternatives for conserving local water resources in California. Water (Switzerland). 2016;8(11):497.

[33] Mekonnen M, Hoekstra A. A global assessment of the water footprint of farm animal products. Ecosystems. 2012;15(3):401–15.

[34] Tom MS, Fischbeck PS, Hendrickson CT Energy use, blue water footprint, and greenhouse gas emissions for current food consumption patterns and dietary recommendations in the US. Environment Systems and Decisions. 2016;36(1):92–103.

[35] Martin M, Danielsson L. Environmental implications of dynamic policies on food consumption and waste handling in the European union. Sustainability (Switzerland). 2016;8(3):282.

[36] Vanham D, Hoekstra AY, Bidoglio G Potential water saving through changes in European diets. Environ Int. 2013;61:45–56. 24096041 10.1016/j.envint.2013.09.011

[37] Vanham D, Bidoglio G. The water footprint of agricultural products in European river basins. Environ Res Lett. 2014;9(6):064007.

[38] Vanham D, Bouraoui F, Leip A, Grizzetti B, Bidoglio G Lost water and nitrogen resources due to EU consumer food waste. Environ Res Lett[Internet] 2015;10(8). doi:10.1088/1748-9326/10/8/084008.

[39] Vanham D, Mekonnen MM, Hoekstra AY The water footprint of the EU for different diets. Ecol Indic. 2013;32:1–8.

[40] Capone R, El-Bilali H, Debs P, Lorusso F, Berjan S Food environmental sustainability in Bosnia, Italy and Serbia: water, ecological and carbon footprints. Kovačević D,editor. In: Third International Scientific Symposium “Agrosym 2012”; November 2012, Jahorina (East Sarajevo), Bosnia and Herzegova. p. 74–9.

[41] Hess T, Andersson U, Mena C, Williams A The impact of healthier dietary scenarios on the global blue water scarcity footprint of food consumption in the UK. Food Policy. 2015;50(0):1–10.

[42] Ruiter de H. Water requirements for food assessed at different levels of scale. [dissertation] University of Groningen CIO, Center for Isotope Research; 2012.

[43] Saez-Almendros S, Obrador B, Bach-Faig A, Serra-Majem L Environmental footprints of Mediterranean versus Western dietary patterns: beyond the health benefits of the Mediterranean diet. Environ Health. 2013;12:118. 24378069 10.1186/1476-069X-12-118 PMC3895675

[44] Thaler S, Zessner M, Mayr MM, Haider T, Kroiss H, Rechberger H Impacts of human nutrition on land use, nutrient balances and water consumption in Austria. Sustainability of Water Quality and Ecology. 2013;1–2:24–39.

[45] Vanham D. The water footprint of Austria for different diets. Water Sci Technol. 2013;67(4):824–30. 23306261 10.2166/wst.2012.623

[46] Hadjikakou M, Chenoweth J, Miller G Estimating the direct and indirect water use of tourism in the eastern Mediterranean. J Environ Manage. 2013;114:548–56. 23176807 10.1016/j.jenvman.2012.11.002

[47] Vanham D, Bidoglio G. The water footprint of Milan. Water Sci Technol. 2014;69(4):789–95. 24569278 10.2166/wst.2013.759

[48] Vanham D, del Pozo S, Pekcan AG, Keinan-Boker L, Trichopoulou A, Gawlik BM Water consumption related to different diets in Mediterranean cities. Sci Total Environ. 2016;573:96–105. 27552733 10.1016/j.scitotenv.2016.08.111

[49] Vanham D, Gawlik BM, Bidoglio G Food consumption and related water resources in Nordic cities. Ecol Indic. 2017;74:119–29.

[50] Vanham D, Mak TN, Gawlik BM Urban food consumption and associated water resources: the example of Dutch cities. Sci Total Environ. 2016;565:232–9. 27173841 10.1016/j.scitotenv.2016.04.172

[51] Djanibekov N, Frohberg K, Djanibekov U Income-based projections of water footprint of food consumption in Uzbekistan. Global Planet Change. 2013;110:130–42.

[52] Harris F, Green R, Joy EJ, Kayatz B, Haines A, Dangour A The water use of Indian diets and socio-demographic factors related to dietary blue water footprint. Sci Total Environ. 2017;587–588:128–36. 10.1016/j.scitotenv.2017.02.085 PMC5378197 28215793

[53] Mukuve FM, Fenner RA. The influence of water, land, energy and soil-nutrient resource interactions on the food system in Uganda. Food Policy. 2015;51:24–37.

[54] Lyakurwa FS. Quantitative modeling of the water footprint and energy content of crop and animal products consumption in Tanzania. Independent Journal of Management and Production. 2014;5(2):511–26.

[55] Yoo SH, Lee SH, Choi JY, Im JB Estimation of potential water requirements using water footprint for the target of food self-sufficiency in South Korea. Paddy Water Environ. 2016;14(1):259–69.

[56] Jalava M, Guillaume J, Kummu M, Porkka M, Siebert S, Varis O Diet change and food loss reduction: what is their combined impact on global water use and scarcity?. Earth's Future. 2016;4(3):62–78.

[57] Jalava M, Kummu M, Porkka M, Siebert S, Varis O Diet change—a solution to reduce water use?. Environ Res Lett[Internet] 2014;9(7). doi:10.1088/1748-9326/9/7/074016.

[58] Damerau K, Patt A, van Vliet O Water saving potentials and possible trade-offs for future food and energy supply. Global Environ Change. 2016;39:15–25.

[59] Davis K, Gephart J, Emery KA, Leach A, Galloway J, D'Odorico P Meeting future food demand with current agricultural resources. Global Environ Change. 2016;39:125–32.

[60] Kummu M, de Moel H, Porkka M, Siebert S, Varis O, Ward PJ Lost food, wasted resources: global food supply chain losses and their impacts on freshwater, cropland, and fertiliser use. Sci Total Environ. 2012;438:477–89. 23032564 10.1016/j.scitotenv.2012.08.092

[61] Godfray HCJ, Aveyard P, Garnett T, Hall JW, Key TJ, Lorimer J, Pierrehumbert RT, Scarborough P, Springmann M, Jebb SA Meat consumption, health, and the environment. Science. 2018;361(6399):eaam5324. 30026199 10.1126/science.aam5324

[62] Dror DK, Allen LH. The importance of milk and other animal-source foods for children in low-income countries. Food Nutr Bull. 2011;32(3):227–43. 22073797 10.1177/156482651103200307

[63] Willett W, Rockström J, Loken B, Springmann M, Lang T, Vermeulen S, Garnett T, Tilman D, DeClerck F, Wood A Food in the Anthropocene: the EAT-Lancet Commission on healthy diets from sustainable food systems. Lancet. 2019;393(10170):447–92. 30660336 10.1016/S0140-6736(18)31788-4

[64] Taylor RG, Scanlon B, Döll P, Rodell M, Van Beek R, Wada Y, Longuevergne L, Leblanc M, Famiglietti JS, Edmunds M Ground water and climate change. Nat Clim Chang. 2013;3(4):322.

[65] De Fraiture C, Wichelns D Satisfying future water demands for agriculture. Agric Water Manage. 2010;97(4):502–11.

[66] Fisher JB, Melton F, Middleton E, Hain C, Anderson M, Allen R, McCabe MF, Hook S, Baldocchi D, Townsend PA The future of evapotranspiration: global requirements for ecosystem functioning, carbon and climate feedbacks, agricultural management, and water resources. Water Resour Res. 2017;53(4):2618–26.

[67] FAO. FAOSTAT food balance sheets. 1961–2013, 2017; [Internet]. Available from: http://www.fao.org/faostat/en/#data/FBS/metadata.

[68] Mekonnen MM, Hoekstra AY. The green, blue and grey water footprint of crops and derived crop products. Hydrol Earth Syst Sci. 2011;15(5):1577–600.

[69] Del Gobbo LC, Khatibzadeh S, Imamura F, Micha R, Shi P, Smith M, Myers SS, Mozaffarian D Assessing global dietary habits: a comparison of national estimates from the FAO and the Global Dietary Database. Am J Clin Nutr. 2015;101(5):1038–46. 25788002 10.3945/ajcn.114.087403 PMC4409685

[70] Ridoutt BG, Sanguansri P, Freer M, Harper GS Water footprint of livestock: comparison of six geographically defined beef production systems. Int J Life Cycle Assess. 2012;17(2):165–75.

[71] Wada Y. Modeling groundwater depletion at regional and global scales: present state and future prospects. Surv Geophys. 2016;37(2):419–51.

[72] Siegfried T, Bernauer T, Guiennet R, Sellars S, Robertson AW, Mankin J, Bauer-Gottwein P, Yakovlev A Will climate change exacerbate water stress in Central Asia?. Clim Change. 2012;112(3-4):881–99.

[73] Ladha JK, Rao AN, Raman AK, Padre AT, Dobermann A, Gathala M, Kumar V, Saharawat Y, Sharma S, Piepho HP et al. Agronomic improvements can make future cereal systems in South Asia far more productive and result in a lower environmental footprint. Global Change Biol. 2016;22(3):1054–74. 10.1111/gcb.13143 26527502

[74] Davis KF, Chiarelli DD, Rulli MC, Chhatre A, Richter B, Singh D, DeFries R Alternative cereals can improve water use and nutrient supply in India. Science Advances. 2018;4(7):eaao1108. 29978036 10.1126/sciadv.aao1108 PMC6031371

[75] Kayatz B, Harris F, Hillier J, Adhya T, Dalin C, Nayak D, Green RF, Smith P, Dangour AD “More crop per drop”: exploring India's cereal water use since 2005. Sci Total Environ. 2019;673:207–17. 30986680 10.1016/j.scitotenv.2019.03.304 PMC6510970

[76] Krishna Bahadur KC, Dias GM, Veeramani A, Swanton CJ, Fraser D, Steinke D, Lee E, Wittman H, Farber JM, Dunfield K When too much isn't enough: does current food production meet global nutritional needs?. PLoS One. 2018;13(10):e0205683. 30352069 10.1371/journal.pone.0205683 PMC6198966

[77] Goldstein B, Hansen SF, Gjerris M, Laurent A, Birkved M Ethical aspects of life cycle assessments of diets. Food Policy. 2016;59:139–51.

