# Supplementary material for: The Water Footprint of Diets: A Global Systematic Review and Meta-analysis
Source: Adv Nutr. 2019 Sep 6;11(2):375–86. doi: 10.1093/advances/nmz091 (PMC7442390; doi:10.1093/advances/nmz091)
Supplement: nmz091_Supplemental_File [file nmz091_supplemental_file.pdf]

Supplemental Table 1 Database specific search strategies used find relevant articles for inclusion in the systematic review.

**Web of science**		
**Set**	***Search***	**Hits (07/02/18)**
# 5	#4 AND #3	3,809
# 4	TS=(diet* OR ((food) NEAR/1 (consumption OR choice* OR secur* OR guideline OR recommendation)))	715,996
# 3	#2 OR #1	121,214
# 2	TS = ((water OR fresh-water OR freshwater OR groundwater OR ground-water OR blue-water OR green-water) NEAR/1 (footprint* OR overconsumpt* OR over-consumpt* OR consumption OR sustainability OR efficien* OR conservation OR saving* OR reduc* OR usage OR resourc* OR security OR availab* OR scarc*))	120,0986
# 1	TS= ("virtual water" OR waterfootprint)	854
**Ovid Medline**		
**Set**	***Search***	**Hits (07/02/18)**
**1**	((water or fresh-water or freshwater or groundwater or ground-water or blue-water or green-water) adj1 (footprint* or overconsump* or over-consump* or consump* or sustainab* or efficien* or conserv* or saving* or reduc* or usage or resourc* or security or availab* or scarc*)). ab, ti.	12550
**2**	("virtual water" or waterfootprint).ab,ti.	120
**3**	1 or 2	12616
**4**	diet*.mp. or (food adj1 (consumption or choice* or secur* or guideline* or recommendation*)).ab,ti.	603894
**5**	3 and 4	1214
**6**	Limit 5 to yr="2000-Current"	748
**Agris OVID**		
**Set**	***Search***	**Hits (07/02/18)**
**1**	((water or fresh-water or freshwater or groundwater or ground-water or blue-water or green-water) adj1 (footprint* or overconsump* or over-consump* or consump* or sustainab* or efficien* or conserv* or saving* or usage or resourc* or security or availab* or scarc*)).ab,ti. (26595)	**26595**
**2**	("virtual water" or waterfootprint).ab,ti.	**154**
**3**	1 or 2	**26625**
**4**	((diet or food) adj1 (consum* or choic* or secur* or guideline* or recommendation*)).ab,ti.	16747
**EconLit OVID**		
**Set**	***Search***	**Hits (07/02/18)**
5	3 and 4	457
# ▲	Searches	Results
1	((water or fresh-water or freshwater or groundwater or ground-water or blue-water or green-water) adj1 (footprint* or overconsumpt* or over-consumpt* or consumption or sustainability or efficien* or conservation or saving* or reduc* or usage or resourc* or security or availab* or scarc*)).ab,ti.	2803
2	("virtual water" or waterfootprint).ab,ti.	67
3	1 or 2	2813

4	diet*.mp. or (food adj1 (consumption or choice* or secur* or guideline or recommendation)).ab,ti. [mp=heading words, abstract, title, country as subject]	5047
5	3 and 4	78
**CAB Abstracts**		
**Set**	***Search***	**Hits (07/02/18)**
**1**	((water or fresh-water or freshwater or groundwater or ground-water or blue-water or green-water) adj1 (footprint* or overconsump* or over-consump* or consump* or sustainab* or efficien* or conserv* or saving* or usage or resourc* or security or availab* or scarc*)).ab,ti. (26595)	**83145**
**2**	("virtual water" or waterfootprint).ab,ti.	**495**
**3**	1 or 2	**83219**
**4**	((diet or food) adj1 (consum* or choic* or secur* or guideline* or recommendation*)).ab,ti. ()	50894
5	3 and 4	1675
**SCOPUS**		
	Search	
	TITLE-ABS-KEY(((virtual water) OR waterfootprint OR ((water OR fresh-water OR freshwater OR groundwater OR ground-water OR blue-water OR green-water) W/1 (footprint* OR overconsumpt* OR over-consumpt* OR consumption OR sustainability OR efficien* OR conservation OR saving* OR reduc* OR usage OR resourc* OR security OR availab* OR scarc*))) AND (diet* OR (food W/1 (consumption OR choice* OR secur* OR guideline OR recommend*))))	**Total hits (07/02/18) – 4238**
**GREENFILE**		
	Search	**Hits (07/02/18)**
	((water or fresh-water or freshwater or groundwater or ground-water or blue-water or green-water) N1 (footprint* or overconsumpt* or over-consumpt* or consumption or sustainability or efficien* or conservation or saving* or reduc* or usage or resourc* or security or availab* or scarc*)) OR ("virtual-water" or waterfootprint) – AB	
	AND	
	diet* or (food N1 (consumption or choice* or secur* or guideline or recommendation)) - AB	
	From 2000	**292**

Supplemental Table 2 Categories of dietary patterns used in the meta-analysis

	Categories used in meta-analysis				
	**Average**	**Healthy**	**Reduced animal source foods**	**No animal source foods**	**Other**
Name of dietary pattern in included studies[1]	Reference<br>Current<br>Total<br>Baseline	National dietary guidelines (USDA, German Nutrition Society)<br>Current + additional protein to meet demand<br>Replaced foods + additional protein<br>Macro-nutrient shift + additional protein and replaced foods<br>Minimum optimised for carbon +nutrient requirements<br>Minimum optimised for nitrogen + nutrient requirements<br>Minimum optimised for water + nutrient requirements<br>Minimum optimised for land + nutrient requirements<br>Minimum optimised for combined environmental impacts + nutrient requirements<br>Dietary guidelines but with lower limit of animal products, higher crops<br>Dietary guidelines but with upper limit of animal products, lower crops<br>Average with reduced kcal<br>Dietary guideline but no change in kcal<br>Dietary guideline + energy reduction<br>Combination of healthy and vegetarian<br>Turkish food based dietary guidelines<br>WHO recommended guidelines<br>Mediterranean dietary pattern	meat 75%, vegetables 200%<br>meat 30%, vegetable 260%<br>meat 50%, vegetables 400%<br>vegetarian<br>healthy pescatarian<br>healthy vegetarian	Vegan<br>Recommended diet with 0% protein from animal sources	Tourist; meat rich, western, holiday diet.<br>FAO recommended calorie level for food security<br>minimum food requirement adjusted to match culturally appropriate foods<br>western pattern<br>European high end tourist<br>European tourist, economy tour<br>European, family travel<br>European, backpacker/eco tour<br>Asian, high end tourist<br>Asian, economy tour<br>Asian, family travel<br>Asian, backpacker/eco tour

[1]Values represent terminology used in the included study

Supplemental Table 3 Studies assessing dietary water use through other metrics (not the water footprint) and therefore were not included in the review

Authors, year (Supp. Ref.)	General study aims	Scale of estimate(s)	Location of estimate(s)	Dietary data source and scenarios (if any)	Water assessment method and data source	Indicator terms used	Findings relevant to this review	Assumptions about imported food
Amarasinghe, U. A. et al. 2007 (69)	Quantify current (2000) and future (2025 and 2050) water use of food consumption in India.	National	India	FAO Food Balance Sheets, food available for supply and data from the National Sample Survey of India.	Calculated from national data	Consumptive water use	Consumptive water use at 567.2 km^3/year for the country. The irrigated crops account for 54% of the total consumptive water use.	Food produced/ consumed in the same area
Chahed, J. et al.2015 (70)	Assess the water equivalent of food stuffs production, trade and demand in Tunisia.	National	Tunisia	Not clear	Modelled based on water use data.	Virtual water content. Food demand water equivalent	The water equivalent of food demand has increased from 1000 m^3/year per capita in the early 1970s to more than 1500 m^3/year per capita in the last 2000s.	Not clear
Chahed, J. et al. 2008 (71)	Assesses the water supply and demand in Tunisia (1990-1997)	National	Tunisia	Not clear	Modelled based on water use data.	Equivalent water for food demand	The equivalent-water for food requirement (11.8 billion m^3/year) is about 1300 m^3/year per capita.	Not clear
Du, B. et al. 2015 (72)	Assesses the direct and indirect water requirements for food consumption from 1995 to 2010 at the household level in the Inner Mongolia Autonomous Region of China.	Sub-national	Hulun Buir, Xilin Gol, and Ordos districts, Northern China.	Food consumption data collected from 209 households in three sub-regions of area.	Based on other sources: Gerbens-Leenes, P.W. and Nonhebel, S. (73);. Li, L. and Wu, X. (74) , Xu, Z.. et al. (75)	Virtual water content	In 1995, the respective virtual water contents of food consumption for Hulun Buir, Xilin Gol and Ordos were; 1758.8 m^3/year per capita, 2377.6 m^3/year per capita and 1838.5 m^3/year per capita, compared to 2307.3 m^3/year per capita, 2054.3 m^3/year per capita and 1553.8 m^3/year per capita in 2010. The virtual water content decreased in the Xilin Gol and Ordos due to decreasing consumption of meat and increasing fruit and vegetables.	Not clear
Gerten, D. et al. 2011 (76)	Assesses global blue and green water availability and corresponding water requirements of current (average between 1972 to 2000) and future (2070-99) food production.	Global/National	Global (all countries)	Scenario diet of 3000 kcal, with 20% animal and 80% vegetal products.	Calculated using the Lund–Potsdam–Jena managed Land (LPJmL) model, that simulates plant growth, production and phenology.	Green and blue water requirements	The global average requirement is 1095 m^3/year per capita, but this varies depending on location; with the lowest in Europe, North America and China. The higher values were in North and East Africa and south-western Asia, countries requiring >2500 m^3/year per capita.	Food produced/ consumed in the same area

Goldstein, B. et al. 2016 (77)	Applies Life Cycle Assessment (LCA) methodology to compare vegetarian and vegan diets to the average Danish diet.	National	Denmark	Average Danish diet from Danish consumption surveys from 2003 to 2008(78). Vegetarian diets were based on the Vegetarian food guide pyramid (Loma Linda University – School of Public Health, 2008. The Vegetarian Food Pyramid (79)).	Taken from the LCA Ecoinvent 3.1 database (consequential modelling) (34).	Water scarcity index	The water scarcity index of the average diet was 0.803 m^3/d per capita, compared to 1.116 m^3/d per capita for vegetarian and 1.117 m^3/d per capita for vegan diets.	Not clear
Kummu, M. et al. 2014 (80)	Compares the effects of hydro climatic variability on the global green and blue water availability and requirements for food production (per food production units) (1977-2006).	Global/Multi-country	Food production units globally	Scenario diet of 3000 kcal/d per capita, with 20% animal and 80% vegetal products.	Calculated using the Lund–Potsdam–Jena managed Land (LPJmL) model that simulates plant growth, production and phenology.	Green and blue water requirements, green and blue water scarcity (based ratio of availability and requirements).	Green and blue water requirements of a reference diet is lowest in in western Europe and some of North America (<650 m^3/year per capita). The requirements are highest (>1300 m^3/year per capita) in northern parts of Latin America, Africa and Southern Asia. Green-blue water scarcity (when requirements are greater than availability) was experienced by 34% of the global population (year 2000). This is mostly found in the Middle East to South Asia.	Food produced/ consumed in the same area
Liu, J. and Savenije, H. H. G. 2008 (81)	Calculates the per capita water requirements for food in China from 1961 to 2003.	National	China	FAO Food balance Sheets, and two scenarios - basic (assuming energy requirements are met by wheat only), and subsistence (based on recommended food intake from the Chinese Nutrition Society (47))	Various sources: Liu, J. and Zehnder, A. et al. (82) Zimmer, D. and Renault, D. (83), and Hoekstra and Chapagain (26).	water requirement, virtual water content	The total water requirement of food was 1127km^3/year for China. The per capita water requirement in 2003 was roughly 860 m^3/year per capita according to FAO food supply accounts, compared to 300 m^3/year per capita for the basic diet, and between 505-730 m^3/year per capita for the subsistence diet (depending on upper and lower boundaries of the recommended daily intake of food).	Food produced/ consumed in the same area
Marlow, H. J. et al. 2015 (84)	Compares the environmental impacts of two dietary patterns in California: higher and lower animal products.	Sub-national	USA (California)	Adventist Health Study (n=34198). Two dietary groups were defined based on their consumption of meat (lower consumption <1 serving of meat/week).	Cost and Return Studies (CRS) published by the University of California Cooperative Extension Service and the University of California Davis Department of Agriculture and Resource Economics	Irrigation rate, irrigation use	The higher animal product diet required 13,545L of water, compared to 3292L for the lower animal product diet (per week).	Food produced/ consumed in the same area
Notarnicola, B. et al. 2017 (85)	Carries out a full life cycle assessment of the average food consumption of a	Multi-country	EU27	Eurostat and FAO databases to develop a "food basket" of representative food products consumed by the average EU27 citizen.	Not clear	Water resources depletion	An average EU citizen incurs 44 m^3/year per capita of water depletion. This could be reduced if animal source food consumption in	Considers import quantity and source from the Eurostat international

	citizen in the European Union 27 Countries.						the diet was reduced by 25 and 50% (estimates in graph).	trade database (2010).
Porkka, M. et al. 2016 (86)	Historical analysis assessing green and blue water requirements globally in each food production unit (from 1905 to 2005).	Global/Multi-country	Food production units globally	Scenario diet of 3000 kcal/d per capita, with 20% animal and 80% vegetal products.	Calculated using the Lund–Potsdam–Jena managed Land (LPJmL) model, that simulates plant growth, production and phenology.	Green and blue water requirements, green and blue water scarcity (based ratio of availability and requirements).	The green-blue water requirements of diets have been decreasing worldwide due to increase in yields. Green-blue water requirements were highest in Central and Southern Africa, Central America and South Asia. By 2005, green-blue water scarcity in terms of available supply to dietary requirements effected 34% of the population.	Food produced/ consumed in the same area
Renault, D. and Wallender, W. W. 2000(87)	Assesses the nutritional water productivity of different crops and animal products, and applies this to the average diet in the USA (1995), comparing different dietary changes.	Sub-national	USA	FAO Food Balance Sheets, and six scenarios for change the water requirements - animal products reduced by 25%, replaced with veg 50% beef replaced with poultry and adjustment of veg 50% red meat replaced with veg Animal products reduced by 50% and replaced with “Vegetarian Survival” - only four products, used to achieve necessary nutrition targets balanced	Calculated (using US statistics and the FAO CROWAT data for reference evapotranspiration(25))	water requirement, water productivity, nutritional water productivity	The average diet of a USA citizen has a water requirement of 5.4 m^3/d. The water productivity increases as the amount of animal source foods decreases. A diet based on survival only (i.e. only using four nutrient rich products), can a water requirement of only 1.0m3 of water per day.	Food produced/ consumed in the same area
Rockström, J. et al. 2007 (88)	Calculates the additional water required to satisfy global hunger targets of the Millennium Development Goals in 92 developing countries.	Multi-country	Developing countries	Current levels based on FAO food balance sheets, but the scenario of a target diet is based on 3000kcal/d per capita with 20% animal and 80% vegetal.	Calculated based on FAO/UN databases.	water productivity, water requirements	To produce a balanced diet, an average pf 1300 m^3/year per capita is needed of freshwater. If water productivity does not improve, and additional 2200km^3/year of vapour flow is needed to halve hunger by 2015 (from 2002 levels).	Not clear
Singh, A. K. et al. 2007 (89)	Assesses the irrigation water requirement in a community of the Mahi (river) command area, and uses linear programme model to reduce the demand while ensuring the minimum requirement for food is met.	Sub-national	Baswara District, Rajasthan, India	Scenario of food requirements based on maize, gram, mustard, wheat and vegetables.	Data collected on irrigation use and environmental conditions in the area.	irrigation water requirement	1420.3 ha m of irrigation water (40% of available water) is required to produce the minimum food required.	Food produced/ consumed in the same area

Supplemental Table 4 Characteristics and results of included studies

Study (Supplemental Reference)	General study aims	Scale of estimate(s)	Location of estimate(s)	Dietary data source and scenarios (if any)	Water assessment method and data source	Indicator terms used	Findings relevant to this review	Assumptions about imported food	Quality level
Birney, C. I et al. 2017 (1)	Quantify environmental impacts of diets in USA (2010) including food loss and waste, and assess changes if diets shifted to those recommended.	National	USA	Uses the Economic Research Service (ERS) Loss-Adjusted Food Availability (LAFA) dataset for food consumption, and the US Department of Agriculture (USDA) dietary guidelines as a scenario	Green and blue water footprints using data from WaterStat and Tom et al., 2016 (2)	Green and blue water footprints	Blue and green water footprints of current dietary patterns are 756400 L/year per capita and 101800 L/year per capita respectively. Shifting to USDA guidelines results in green WFs 699700 L/year per capita, blue WF 114000 L/year per capita. The amount of food is only available in kcal/d per capita so couldn't include in quantitative analysis.	Food produced/ consumed in the same area, and global average water footprints applied if USA was not available.	high
Blas, A. et al. 2016 (3)	Composed seasonal menus of the recommended Mediterranean and the USDA diets, and compared WFs of each if produced in Spain vs USA.	National	Spain, USA	Scenario diets; Mediterranean Diet Foundation, US Department of Agriculture	Water Footprint Assessment Method, WaterStat database	Green, blue and grey water footprints	Mediterranean dietary pattern has lower WF in both countries, compared to the USDA. The WF of Mediterranean diet in Spain is 5276 L/d per capita, switching to USDA would increase this to 6870 L/d per capita - mainly due to increased green water use. The USDA WF in the US is 5632 L/d per capita, switching to the Mediterranean would result in a decreased WF of 4003 L/d per capita.	Considers imports, but only for some products and assuming weighted average from import countries (FAOStat trade matrix (4))	high
Capone, R. 2012 (5)	Compares water footprints, carbon footprints and ecological footprints between the three different countries based on 2006.	National	Italy, Bosnia, Serbia	FAO Food balance Sheets, food available for supply	Water Footprint Assessment Method, WaterStat database	Green, blue and grey water footprints	The total green and blue water footprints of food supply were similar in Bosnia and Italy (1686.01 Million m^3 and 1683.4 Million m^3 respectively), and highest in Serbia. Meat is the highest contributor to	Considers imports, weighted based on origin (data source not clear).	medium

							the water footprint in all three countries.		
Damerau, K. et al. 2016 (6)	Investigates current (2011) and future environmental impacts (2050), based on changes to food preferences and fuel use. Explores dietary change scenarios by increase protein to match demand and substituting items.	Multi-country	Regions; Asia, Latin America, Middle East, OECD, Eastern Europe and Soviet Union	FAO Food Balance Sheets; food available for supply. Dietary change scenarios were; increasing protein supply to match the level in OECD countries, swapping certain foods while maintaining macro-nutrient share, and decreasing carbohydrate in the diet while substituting with fat.	Water Footprint Assessment Method, WaterStat database	Blue water footprints, Water intensity	Blue water footprints are lowest in the Middle East and Africa (481 L/d per capita), and highest in the Eastern European and Soviet Union (992 L/d per capita) and Asia (751 L/d per capita). In all regions, altering the macro-nutrient content of the diet (to more protein) and replacing certain foods (for example cereals, dairy) with less water demanding products (e.g. tubers, eggs), results in reduced blue water footprint.	Food produced/ consumed in the same area	high
Davis, K. F. et al. 2016 (7)	Explores environmental impacts current and future diet (2050) and assesses the potential of dietary change scenarios.	Global	Global	FAO Food balance Sheets, food available for supply	Water Footprint Assessment Method, WaterStat database	Total water footprint (green and blue)	776 m^3/y is required to support an average global diet (circa 2009). Animal products contribute to 43% of this.	Food produced/ consumed in the same area	high
Djanibekov, N. et al. 2013 (8)	Quantified the national water footprints of food consumption in Uzbekistan (2009) and projects income driven changes to the population's diet and resulting water footprints to 2034.	National	Uzbekistan	FAO Food balance Sheets, food available for supply	Water Footprint Assessment Method, WaterStat database	Total water footprint (green and blue)	The total water footprint of food consumption in Uzbekistan 1097 m^3/y per capita	Not clear	high
Gephart, J. A. et al. 2016 (9)	Minimise water, nitrogen, carbon and land footprints of diets based on nutritional and population data from the United States.	National	USA	Scenario of minimising environmental impacts while achieving nutritional needs. Food products and groups based on the USDA Dietary Guidelines and the Harvard University Healthy Eating Plate. Scenarios were calculated first with no constraint on the serving number, and second with constraints of maximum of 26 servings of each specific food item.	Water Footprint Assessment Method, WaterStat database, plus an additional estimation of the water footprint of seafood based on Gephart et al., 2014. (10)	Total water footprint (green and blue)	Diets that were optimised for nutrition and water with no constraint of serving number could achieve a total water footprint of 0.62 m^3/d per capita. However, when the 26 serving constraint was added this increased to 2.26 m^3/d per capita. If diets are optimised to all environmental	Food produced/ consumed in the same area	medium

							impacts and nutrient constraints, the water footprint is 2.46 m^3/d per capita.		
Goldstein, B. et al. 2017 (11)	Applies Life Cycle Assessment (LCA) methodology to assess the potential for a plant-based burger to reduce the environmental impacts of food demand in the United States through vegetarian and vegan diets.	National	USA	USDA loss-adjusted-food-availability estimates (2010) for the average diet, and the vegetarian and vegan diets are based on the USDA's 2010 dietary guidelines.	Ecoinvent 3.2 database	Blue water footprint	The mean US diet has a blue water footprint of 294 m^3/y per capita. Vegetarian and vegan diets would reduce this by 62% and 70% respectively (when diets remain iso-caloric). Substituting ground beef for a plant based burger at 10%. 25% and 50% would also reduce water use by 6 (2.1%), 15 (5.2%) and 31 (10.4%) m^3/y per capita.	Not clear	medium
Hadjikakou, M. et al (12)	Compares the water footprints (direct and indirect) of five different tourist groups travelling from the UK to the Eastern Mediterranean (Cyprus, Turkey, Greece, Syria).	Population group – tourists	Eastern Mediterranean	Scenario diets based on different types of holiday; Luxury golf holiday; meat-rich diet, walking/hiking holiday; vegan diet, budget beach holiday; western diet, relaxing beach holiday; holiday diet, backpacking; local diet.	Water Footprint Assessment Method, WaterStat database	Green and blue water footprints, virtual water content	Diets are the largest component of tourist's water use. Meat contributes to over 75% of the water use for all diets, except the vegan one. However, fruit and vegetables in the vegan diet had a particular high water footprint.	Considers import quantity through FAOStat trade balance sheets (4), WF value assumed to be the same as local.	high
Hai-yang, S. 2015 (13)	Assesses the virtual water content of food consumption in the Gansu province, China (1992-2005), and quantifies the water saving potential of diet changes; reducing meat and increasing vegetables.	Sub-national	China (Gansu)	Gansu Province Statistical Yearbook for average consumption and three scenarios of changing meat and vegetable products.	Water Footprint Assessment Method, WaterStat database	Total water footprint (green and blue)	The average water footprint of an individual in the Gansu province is 698m^3/y per capita. This decreases with reduction in meat; for an iso-caloric diet, the total water footprint is 635m^3/y per capita with 50% reduction in meat and a 400% increase in vegetables.	Not clear	low
Harris, F. et al. 2017 (14)	Quantifies the green and blue water footprints of diets in	National/Sub-National	India	Dietary data from food frequency questionnaire in India (15) (n=6775)	Water Footprint Assessment Method,	Green and blue water footprints	An Indian diet has an average (SD) green water footprint of 2531 (885) L/d	Food produced/ consumed in the same area	high

	India, comparing the blue water footprint between different socio-demographic groups				WaterStat database, with additional adjustments of animal source foods based on the spatial variability in the water footprint of feed.		per capita, and blue of 737 (263) L/d per capita. The blue water footprint is lowest in the Southern region, and highest for urban and wealthier populations.		
Hess, T. et al. 2015 (16)	Calculates the water footprint and blue water scarcity footprint of UK food consumption (2005), and assesses alternative future scenarios dietary scenarios and their effect on global water scarcity.	National	UK	UK food consumption obtained from Audsley et al., 2009 (17)	Blue and green water footprints obtained using Water Footprint Assessment Method, WaterStat database. Virtual blue water scarcity calculated using country specific estimates of Water Stress Index (18)	Blue and green virtual water consumption; Water scarcity footprint	The average total dietary water footprint in the UK 2400L/d per capita, of which 160L/d per capita is blue.	Considers import quantity and water footprint in country of origin, using UK trade data from HM Revenue and Customs, 2013 (19) and INTRACEN, 2013(20).	medium
Jalava, M. et al. 2016 (21)	Quantifies water footprints of national diets globally (2009-2011), and assesses the potential to reduce water use and scarcity by changing diets (recommended and reducing animal source foods) and reducing food loss and waste.	Global/National	Global	Current food consumption based on Food and Agricultural Organisation Food Balance Sheets. Scenarios were changing diets based on WHO recommendations (22), and four diet scenarios with 50%, 25%, 12.5%, and 0% cap on animal based protein, of which one third can be from meat.	Water Footprint Assessment Method, WaterStat database	Green and blue water footprints and water saving	Shifting global diets to those recommended would decreased the blue and green water footprints by 6% and 7% respectively. Reducing animal source foods by 25% would decrease this further; - 11% for blue, -18% for green.	Considers import quantity from FAO trade data (4), uses global average water use values	high
Jalava, M. et al. 2014 (23)	Compares the water footprint of current national diets globally (2007-2009) to diets that follow recommendations and four scenarios of reducing animal sources foods.	Global/National	Global	Current food consumption based on Food and Agricultural Organisation Food Balance Sheets. Scenarios were changing diets based on WHO recommendations(22), and four diet scenarios reducing animal sources foods to 50%, 25%, 12.5% and 0% of the total protein intake.	Water Footprint Assessment Method, WaterStat database	Green and blue water footprints	In regions of the world consuming diets that are excess in energy, the blue water footprint is 360L/d per capita and green 2563L/d per capita. This could be reduced by 6% for green and blue if following the recommended diet, or 19% (blue) and 22% (green) if no animal source protein. In regions of the world that need to increase energy	Considers imports from FAO trade data (4), using water footprint of weighted average of all global exports	high

							intake of the diet, the green water footprint was 1943L/d per capita and blue 442L/d per capita. Switching to the recommended diet would increase the green and blue water footprints by 7%, but reducing animal source foods to 0% decreases the blue water footprint by 8% and green by 17%.		
Kang, J. F. et al. 2017 (24)	Calculates the water footprint of food consumption in rural and urban Xiamen, China, and uses decomposition analysis to assess the driving forces in water footprint change (2001-2012).	Sub-national	China (Xiamen)	Food consumption data from the Yearbook of Xiamen Special Economic Zone (2002-2013)	Followed the Water Footprint Assessment method, using CROPWAT software (25) for local crops and Hoekstra and Chapagain (26) for imported foods and livestock.	Total water footprint (green and blue), virtual water content	The total water footprint of food consumption in Xiamen in 2001 was 725 Million m^3/y compared to 1369 Million m^3/y in 2012. For Xiamen city specifically, the food consumption water footprints were 524 Million m^3/year in 2001 compared to 1199 Million m^3/y in 2012. Values could not be converted to per capita for the analysis.	Considers imports in the virtual water content of crops, although methods are not clear.	medium
Kummu, M. et al. 2012 (27)	Estimates the water use for domestic food supply and corresponding food loss and waste for all countries globally.	Global/ Multi-country	Regions: Africa, Europe, Industrialised Asia, Latin America, North Africa & Western-Central Asia, South & Southeast Asia, Global	FAO Food balance Sheets, food available for supply but with additional adjustments for food waste.	Water Footprint Assessment Method, WaterStat database	Blue water footprint	The global average blue water footprint of food supply is 111 m^3/y per capita. It is highest in North Africa & West-Central Asia 258 m^3/y per capita, and lowest in Sub-Saharan Africa at 52 m^3/y per capita.	Considers import quantity, using weighted average of all global exports for water footprint	high
Li, J. 2017 (28)	Assesses the direct and indirect water footprints of tourists in the Beijing-Tianjin-Hebei metropolitan region of China.	Population group – tourists	China	Four scenario diets for different tourist groups (for each Western and Asian); high end, economy, family travel and backpacker.	Water Footprint Assessment Method, WaterStat database	Total water footprint	Western high end tourists have the highest dietary water footprint at 8520 L/d per capita, compared to an Asian backpacker tourist with only 2797 L/d per capita. Included in the analysis as food groups converted from kcal to kg/y per capita based on conversion rates given by author.	Not clear	medium

Lyakurwa, F. S. 2014 (29)	Assesses the water footprint of food consumption in Tanzania, linking the water footprints with energy values of food, and calculates the water savings of different dietary scenarios (reducing animal source foods).	National	Tanzania	FAO Food balance Sheets, food available for supply	Water Footprint Assessment Method, WaterStat database	Water footprint (water saving)	The water saving of dietary scenarios ranges between 688 Million m^3 if 100% of animal products are replaced with vegetable products, compared to 28 Million m^3 if 25% of wheat and rice consumption is replaced with fruits. Baseline dietary water footprint was not available.	Considers import quantity, using FAO food balance sheets. Water use data not clear.	low
Marrin, D.L. 2016 (30)	Estimates the local blue water used for animal and plant-based food, and compares the potential for dietary shifts and reducing food waste of local residents to reduce local blue water use.	Sub-national	USA (California)	Not clear	Obtained from a report undertaken by the Pacific Institute (31)	blue water footprint	Animal based foods consume an average of 7 billion m^3/y compared to 3.1 billion m^3/y in California. Adopting one vegan day per week could decrease the local blue water footprint by 6%, compared to 14% for one vegan meal per day.	Food produced/ consumed in the same area	low
Martin, M. and Danielsson, L. 2016 (32)	Uses life cycle assessment methodology to calculate the environmental impacts of food consumption in the European Union (2010), and compares policy options for reducing them to 2030 and 2050.	Multi-country	EU27	FAO Food balance Sheets, food available for supply	Ivanova et al., 2015.(33) and the Ecoinvent database (34)	blue water footprint	In 2010, the blue water footprint of EU food consumption as 98700 Million m^3 (including waste figures).	Not clear	low
Mekonnen, M. M. and Hoekstra, A. Y. 2012 (35)	Quantifies the water footprints of animal products globally, and includes an estimate for the water saving if the average American switched to vegetarian or vegan diets.	National	USA	Scenario: replacing all meat with an equivalent amount of crop products (pulses and nuts)	Water Footprint Assessment Method, WaterStat database	Water footprint (water saving)	Meat contributes to 37% of the total dietary water footprint of an American. Replacing all meat with plant products decreases the water footprint by 30%.	Not clear	low
Mukuve, F. M. and Fenner. R.A. 2015 (36)	Calculates the current (2012) water resource use of food consumption in Uganda, and assesses the potential water resource use to achieve food security (in 2012, and 2050).	National	Uganda	FAO Food balance Sheets, food available for supply (1900 kcal/d per capita), and a scenario for increasing Uganda food consumption to FAO's recommended daily calorie intake level of 3000 kcal/d per capita (37).	Based on diet for Sub-Saharan Africa from Rockström, 2003 (38).	Total water footprint (green and blue)	The current diet results in the water consumption of 690 m^3/y per capita, compared to 1300 m^3/y per capita if daily calorie needs are met.	Not clear	low

Ruiter de, H. 2012 (39)	Assesses the potential to reduce water use by quantifying the water use of the food production system at different levels (e.g. crop, agricultural and cultural).	National	The Netherlands, Spain	Scenario diet to match minimum food requirements in The Netherlands and Spain, and a more culturally acceptable diet in The Netherlands.	Calculated water requirements and Water Footprint Assessment Method, WaterStat database	Total water footprint (green and blue), water requirements.	The minimum amount of water required to produce a diet (consisting of sugar beet, rapeseed and oats) is 295 L/d in the Dutch system, however the location of production matters where it would be 686 L/day per capita. If diets consist of the four most eaten foods in The Netherlands, the water requirements increase to 1413 L/day per capita.	Food produced/ consumed in the same area	low
Saez-Almendros, S. et al. 2013 (40)	Compares the environmental impacts of the current Spanish diet to the Mediterranean Diet Pattern and an average USA (Western) diet.	National	Spain	For current consumption, uses FAO Food Balance Sheets (2007) and the Household Consumption Surveys of the Spanish Ministry of Agriculture, Food and Environment (6000 households). Scenarios are Western (USA - FAO FBS) diet, and a diet based on the Mediterranean Diet Pattern Pyramid.	Various sources: Water Footprint Assessment Method, WaterStat database., Eurostat database, Garrido et al., 2012 (41). Gazulla et al., 2010 (42).	Total water footprint (green and blue)	The average diet of a Spanish citizen has a total water footprint of is 19.7 km^3/y if FBS are used to quantify consumption, compared to 13.4 km^3/y with household consumption surveys. The MDP has a water footprint lower at 13.3 km^3/y, but the WDP is highest at 22.0 km^3/y.	Food produced/ consumed in the same area	high
Song, G et al. 2015 (43)	Quantifies the environmental impacts of food consumption and waste of a household in China.	National	China	Chinese Health and Nutrition Survey database (2004-2009)	DEFP database from the Barilla foundation	Total water footprint (green and blue)	The average household in China has a dietary water footprint of 2436 m^3/y, which equates to 673 m^3/y per capita.	Not clear	medium
Sun, S et al. 2015 (44)	Calculates the water and energy conversion efficiencies of different crops in China, and assesses water saving potential through changing food consumption in China.	National	China	Chinese statistical year book (2011) (45) and China Agriculture Statistical Report (2011) (46) for current consumption, and scenario diets based on lower and upper limits from the Dietary Guidelines for Chinese Residents, 2011 (47).	Water Footprint Assessment Method, WaterStat database	green and blue water footprints	If diets in China were adjusted to healthy dietary guidelines, this could achieve a green water saving of between -59.79 Gm^3 (for lower limit of animal source foods), while the blue water footprint could decrease by 4.64 Gm^3. If diets were shifted to the upper limit of animal source foods in the dietary guidelines, this would increase water use by 0.11 Gm^3.	Food produced/ consumed in the same area	low

Thaler, S. et al. 2014 (48)	Undertakes an environmental impact assessment of food consumption in Austria (2001-2006)	National	Austria	Statistik Austria, 2007 (49)	Used the Water Footprint Assessment but calculated based on available data in Austria.	green and blue water footprints	The green water footprint was 3.9 m^3/d per capita, and blue was 0.04 m^3/d per capita. Animal source foods are responsible for 87% of the total water footprint.	Considers import quantity from Statistik Austria 2007 supply balance accounts, using global average water footprints	high
Tom, M. S. et al. 2016 (2)	Compares the potential to reduce environmental impacts of USA food consumption through different dietary strategies.	National	USA	Calculated based on US Department of Agriculture and US Department of Health and Human Services 2010 data, and total energy intake based on calculated requirements from the National Health and Nutrition Examination Survey. The three dietary scenarios include 1) reducing calories to sufficient level, 2) changing food mix to patterns recommended by the USDA Dietary Guidelines, without reducing Caloric intake, and 3) reducing Caloric intake levels and shifting food mix to meet USDA Dietary Guidelines.	Water Footprint Assessment Method, WaterStat database	blue water footprints	Compared to current average intake, shifting to healthier diets in the USA would result in an increased blue water footprint by around 16%. Reducing caloric level to proposed level for normal weight would decrease the blue water footprint by around 9%. Combination of both changing the food mix and reducing calories increases the water footprint by 10%.	Food produced/ consumed in the same area	medium
Vanham, D. 2013 (50)	Analysis the water footprint of current diets in Austria and compares to healthier and vegetarian diets.	National	Austria	Current food intake based on FAO FBS, with conversion factors applied to account for waste and other uses (Statistics Austria data, Zessner et al., 2011 (51)	Water Footprint Assessment Method, WaterStat database	green and blue water footprints	The green water footprint was 3108 L/d per capita, and the blue was 181 L/d per capita. Dietary scenarios could not be used as they contain grey water.	Not clear	high
Vanham, D. et al. 2014 (52)	Compares the water footprint of the average diet in the EU28 (EU27+Croatia), to a healthy diet, vegetarian and combined diet.	Multi-country	EU28	For current consumption, uses FAO Food Balance Sheets (1996-2005), with additional conversion factors for waste and other uses (51, 53) Recommended diet based on the German Nutrition Society recommendation; healthy, vegetarian, combined.	Water Footprint Assessment Method, WaterStat database	green and blue water footprints	The water footprint of the reference diets in the EU28 had a green water footprint of 3572 L/d per capita, and a blue of 299 L/d per capita. Healthier diets had lower water footprints than the reference, but vegetarian diets had the lowest green and blue water footprints (2187 and 206 L/d per capita respectively).	Not clear	medium
Vanham, D. and Bidoglio, G. 2014 (54)	Assesses the agricultural water footprints in 365 European river basins, and compares this to two dietary scenarios; healthy and vegetarian.	Multi-country/National	Europe	FAO Food Balance Sheets, food available for supply for current consumption. Healthy dietary scenarios were based on regional FBDG for the 40 nations separately.	Water Footprint Assessment Method, WaterStat database	green and blue water footprints	If diets were to shift to healthier patterns, this would decrease the water footprints in most river basins (max -32%), however it increased in some areas	Not clear	medium

							such as northern and eastern Europe.		
Vanham, D. and Bidoglio. D. 2014 (55)	Quantifies the water footprint of Milan, including agricultural, industrial and domestic use.	Sub-national	Italy (Milan)	FAO Food balance Sheets, food available for supply for current consumption, as well as Mediterranean dietary guideline (56) for a healthy diet and vegetarian diet.	Water Footprint Assessment Method, WaterStat database	green and blue water footprints	The current diets in Milan have a green water footprint of 4714 L/d per capita and a blue of 441 L/d per capita. By switching to healthier diets this could be reduced to; green 3196 L/d per capita, blue 321 L/d per capita. This is even more for vegetarian diets; green: 2592 L/d per capita, blue: 280 L/d per capita	Not clear	medium
Vanham, D. et al. 2015 (57)	Calculates the water and nitrogen use of EU food consumption and waste.	Multi-country	EU	FAO Food Balance Sheets, food available for supply for current consumption, with correction factors applied for waste and other uses.	Water Footprint Assessment Method, WaterStat database	green and blue water footprints	The green water footprint of EU food consumption was calculated at 3383 L/d per capita, and the blue was 270 L/d per capita.	Food produced/ consumed in the same area	high
Vanham, D. et al. 2016.(58)	Estimates the water footprints associated with food consumption in 13 Mediterranean cities (1995-2005) and assesses the potential for different dietary strategies to reduce this (healthy with meat, healthy pescatarian, healthy vegetarian).	Sub-national	Croatia (Dubrovnick), France (Lyon), Greece (Athens), Israel (Jerusalem), Italy (Genova, Pisa, Bolgona, Reggio), Slovenia (Ljublijana), Spain (Manresa, Zaragoza), Turkey (Istanbul, Ankara)	FAO FBS with correction factors (using national surveys for each country), and scenarios for reducing water footprints. Healthy meat patterns all based on the Mediterranean diet (56).	Water Footprint Assessment Method, WaterStat database	green and blue water footprints	The total water footprints of current food consumption ranged from 3277 L/d per capita in Ljubljana, to 5789 L/d per capita. in Jerusalem. Switching to a healthy diet could reduce this in all cities, with the healthy vegetarian diets having the lowest total water footprints (2211 L/d per capita in Ljubljana).	Not clear	high
Vanham, D. et al. 2017.(59)	Quantifies the water footprint the direct and indirect water footprints in Hong Kong (1995-2005) and compares the water footprint of different dietary scenarios (current, healthy, pescatarian, and vegetarian).	Sub-national	China (Hong Kong)	FAO FBS with correction factors (for food use and waste), and dietary scenarios based on recommendations from the Chinese Nutrition Society (47), with adjustments for calorie requirements based on the population distribution. Pescatarian was healthy but with all meats/animal fats substituted for plant products, vegetarian is healthy but with all fish and meats substituted for plant products.	Water Footprint Assessment Method, WaterStat database	green and blue water footprints	The total water footprint of diets in Hong Kong was 4727 L/d per capita, of which the blue water footprint was 634 L/d per capita. With healthy dietary shifts, this total water use was reduced by 40%. The largest reduction was achieved from switching to healthy vegetarian diets; a green water footprint of 1832 L/d per capita and a blue of 392 L/d per capita	Considers import quantity and source (FAO trade matrix)(4).	high

Vanham, D et al. 2017 (60).	Calculates the water footprint of food consumption (1995-2005) in different Nordic cities, and assesses the potential for different dietary strategies (healthy, pescatarian, vegetarian) to reduce this.	Sub-national	Sweden (Stockholm, Malmo, Eslov, Helsingborg, Kristianstad), Denmark (Copenhagen), Finland (Helsinki), Norway (Oslo), Iceland (Reykjavik)	FAO FBS with additional calculations using national dietary of food surveys for each country. For the Healthy dietary scenarios, used new Nordic Nutrition Recommendations (NNR) of 2012 (Nordic Council of Ministers, 2012), healthy pescatarian based on the NNR, and healthy veg based on NNR	Water Footprint Assessment Method, WaterStat database	green and blue water footprints	The water footprints vary between 3552 L/d per capita in Denmark to 2865 L/d per capita in Helsinki. Switching to healthy diets reduced the water footprint for all cities. The greatest reduction can be achieved by switching to healthy vegetarian diets (between -35% to -44%).	Not clear	medium
Vanham, D. et al. 2013 (61)	Compares the water footprints of food consumption (1995-2005) between the North, West, South and Eastern EU zones, and calculates the water footprint for healthy and vegetarian diets in each region.	Multi-country	EU - East, North, South, West	FAO FBS with correction factors for current consumption. Healthy dietary scenario is based on regional dietary guidelines (e.g. German Nutrition Society, Mediterranean dietary guidelines).	Water Footprint Assessment Method, WaterStat database	green and blue water footprints	The water footprints of current diets are 5364 L/d per capita (South), 3635 L/d per capita (East), 3421 L/d per capita (West) and 2889 L/d per capita(North). Diets in the South had the highest blue water footprint at 618 L/d per capita. Switching to healthy diets would reduce this between -30 to -3%. Vegetarian diets would reduce total water footprints to between -41% to -27% (depending on region).	Food produced/ consumed in the same area	high
Vanham, D et al. 2016 (62)	Assess the water footprint associated with direct use and food consumption (1995-2005) in Dutch cities with different levels of urbanisation, and compares current dietary water footprint to healthy, pescatarian and vegetarian diets.	Sub-national	The Netherlands (Amsterdam, Dordrecht, Rotterdam, Einhoven, Maastricht, Nieuwegin, Venlo)	FAO FBS, and Dutch National Food Consumption Survey (DNFCS) 2016. The DNFCS was used to distinguish food consumption by urbanisation level. Ref year for FBS 1996-2005. Healthy diets based on Dutch Food Based Dietary Guidelines, pescatarian is the same as healthy but with all meat products replaced with plant products, and vegetarian is all the meat and fish products replaced with plant products.	Water Footprint Assessment Method, WaterStat database	total (green and blue water footprints combined)	The total water footprint of current diets ranged from 3126L/d per capita in strongly urbanised cities to 3245 L/d per capita in extremely urbanised cities. All dietary scenarios explored reduced the water footprint of food consumption, but the lowest values were achieved for vegetarian diets; between 1860L/d per capita for Nieuwegin to 1883L/d per capita for Amsterdam.	Food produced/ consumed in the same area	medium

Yoo, S. H.et al. 2016 (63)	Observed the trends in water footprints over 25 years in South Korea (from 1985 to 2010), future food production and consumption scenarios were explored in 2015 and 2020 for the targets of food self-sufficiency.	National	South Korea	Korea Rural Economic Institute (KREI) (2011) Food balance sheet. Korea Rural Economic Institute, Seoul (in Korean)	Various National databases; Yoo et al., 2014a (64). Yoo et al., 2014b (65), Lee et al., 2015 (66).	green and blue water footprints	The water footprint of food consumption has increased in South Korea from 758.9 m^3/y per capita (1995) to 822.9 m^3/y per capita (2010. In 2010, the green water footprint was 754 m^3/y per capita and the blue was 68.9 m^3/y per capita. Cereals and meats accounted for 18.3 and 38.6 % of the total water footprint of food consumption in 2010.	Not clear	medium
Yuan, Q. et al. 2016 (67)	Assesses the water footprint of food consumption in the Heilongjiang northernmost province of China, comparing the differences between rural and urban households.	Sub-national	China (Heilongjiang)	China Health and Nutrition Survey	Water Footprint Assessment Method, WaterStat database	green and blue water footprints	The average total dietary water footprint in the region was 1.47m^3/d per capita. This was higher in the urban region compared to rural. The green water footprint in the urban area was 1.64 m^3/d per capita, and blue 0.32 m^3/d per capita, in the rural the green was 1.14 m^3/d per capita and the blue was 0.26 m^3/d per capita.	Not clear	low
Zhuo, L. et al. 2016 (68)	Quantifies the consumptive water use and virtual water trade in China from 1978-2008, and considers water use under future scenarios (to 2030 and 2050).	National	China	FAO Food Balance Sheets; food available for supply.	Water Footprint Assessment Method, WaterStat database	total (green and blue water footprints combined)	The total water footprint of Chinese food consumption in 2005 was 927 m^3/y per capita (baseline scenario for the analysis).	Considers import quantity through the difference between production and consumption, and applies global average WFs for the crops.	medium

Supplemental Table 5 Quality scores of included studies

**Study (Supplemental Ref.)**	**Was the baseline diet source stated?**	**Is there a clear description of the baseline diet pattern?**	**Is the full diet assessed?**	**Is there a clear description of the water use assessed?**	**Is the water use data source clearly stated?**	**Is there a clear description of the study area/ population?**	**Is there a description of methods used to link consumption-water (e.g. consideration of trade or other factors)?**	**Are the assumptions/ limitations stated?**	**Are there confidence limits around the estimated dietary water use?**	**For studies assessing scenarios, is there a clear justification/ description of the scenario diet?**	**%**	**quality - >50% = low, 50-70% = medium, >70%= high**
Birney, C. I et al. 2017 (1)	1	1	0	1	1	1	1	1	0	1	80	high
Blas, A. et al. 2016 (3)	1	1	0	1	1	1	1	1	0	1	80	high
Capone, R. 2012 (5)	1	1	0	1	1	1	0	0	0	NA	55.6	medium
Damerau, K. et al. 2016 (6)	1	0	1	1	1	1	1	1	0	1	80	high
Davis, K. F. et al. 2016 (7)	1	1	1	1	1	0	0	1	1	NA	77.8	high
Djanibekov, N. et al. 2013 (8)	1	1	1	1	1	1	0	1	0	NA	77.8	high
Gephart, J. A. et al. 2016 (9)	1	1	0	1	1	0	0	1	0	1	60	medium
Goldstein, B. et al. 2017 (11)	1	1	1	1	0	0	1	1	0	1	70	medium
Hadjikakou, M. et al (12)	1	0	1	1	1	1	1	1	0	NA	77.8	high
Hai-yang, S. 2015 (13)	1	0	1	0	0	1	0	0	0	0	30	low
Harris, F. et al. 2017 (14)	1	1	1	1	1	1	1	1	1	NA	100	high
Hess, T. et al. 2015 (16)	1	0	1	1	1	0	1	1	0	NA	66.7	medium
Jalava, M. et al. 2016 (21)	1	1	1	1	1	1	1	1	0	1	90	high
Jalava, M. et al. 2014 (23)	1	1	1	1	1	0	1	1	0	1	80	high
Kang, J. F. et al. 2017 (24)	1	0	1	1	1	1	1	0	0	NA	66.7	medium

Kummu, M. et al. 2012 (27)	1	0	1	1	1	1	1	1	0	NA	77.8	high
Li, J. 2017 (28)	0	1	1	0	1	0	0	1	0	1	50	medium
Lyakurwa, F. S. 2014 (29)	0	0	1	1	1	0	0	0	0	0	30	low
Marrin, D.L. 2016 (30)	0	0	0	1	0	0	1	0	0	NA	22.2	low
Martin, M. and Danielsson, L.. 2016 (32)	1	1	0	1	1	0	0	0	0	NA	44.4	low
Mekonnen, M. M. and Hoekstra, A. Y. 2012 (35)	0	0	0	1	1	0	0	0	0	0	20	low
Mukuve, F. M. and Fenner. R.A. 2015 (36)	0	0	1	0	1	0	0	0	0	1	30	low
Ruiter de, H. 2012 (39)	1	0	0	0	1	0	0	1	0	1	40	low
Saez-Almendros, S. et al. 2013 (40)	1	0	1	1	1	1	1	1	0	1	80	high
Song, G et al. 2015 (43)	1	1	1	0	1	1	0	0	1	NA	66.7	medium
Sun, S et al. 2015 (44)	1	0	0	0	1	1	0	0	0	NA	33.3	low
Thaler, S. et al. 2014 (48)	1	1	0	1	1	1	1	1	0	NA	77.8	high
Tom, M. S. et al. 2016 (2)	1	0	1	1	1	0	0	1	1	NA	66.7	medium
Vanham, D. 2013 (50)	1	1	1	1	1	1	1	0	0	1	80	high
Vanham, D. et al. 2014 (52)	1	1	1	1	1	0	0	0	0	1	60	medium
Vanham, D. and Bidoglio, G. 2014 (54)	1	0	1	1	1	0	0	0	0	1	50	medium
Vanham, D. and Bidoglio. D. 2014 (55)	1	0	1	1	1	0	0	1	0	1	60	medium
Vanham, D. et al. 2015 (57)	1	1	1	1	1	1	0	1	0	NA	77.8	high
Vanham, D. et al. 2016.(58)	1	1	1	1	1	1	0	1	0	1	80	high
Vanham, D. et al. 2017.(59)	1	1	1	1	1	1	1	1	0	1	90	high
Vanham, D et al. 2017 (60).	1	1	1	1	1	1	0	0	0	1	70	medium

Vanham, D. et al. 2013 (61)	1	1	1	1	1	1	0	1	0	1	80	high
Vanham, D et al. 2016 (62)	1	0	1	1	1	1	0	0	0	1	60	medium
Yoo, S. H.et al. 2016 (63)	1	1	1	1	1	1	0	0	0	NA	66.7	medium
Yuan, Q. et al. 2016 (67)	1	0	0	1	0	0	0	0	0	NA	22.2	low
Zhuo, L. et al. 2016 (68)	1	0	0	1	1	1	1	1	0	NA	66.7	medium

Supplemental Table 6 Major food groups contributing to each dietary WF for the corresponding patterns. N studies = 30. Light colours boxes indicate information was not available.

KEY	mixed animal source and plant based foods	fruits and vegetables	animal source foods	grains, cereals, potatos	other plant based foods

			Main food groups contributing to the dietary water footprint (%)*					
Study (Supplemental Ref.)	Country/Region	Diet pattern	Blue		Green		Total	
Birney et al. 2017 (1)	USA	average	meat, poultry, eggs (24%)	grains (13%)	meat, poultry, eggs (49%)	dairy (15%)		
Capone 2012 (5)	Italy, Bosnia, Serbia	average					meat (beef) (32-42%)	dairy (milk) (10-22%)
Davis et al. 2016 (7)	Global (245 countries)	average					grains (30%)	beef meat (12%)
Djanibekov et al. 2013 (8)	Uzbekistan	average					meat (42%)	wheat (16%)
Goldstein et al. 2017 (11)	USA	average	protein (74%)	grain (10-11%)				
Hai-yang 2015 (13)	China	average					fruits (12-16%(	eggs (8-12%)
Harris et al. 2017 (14)	India	average	wheat (0-88%)	rice (0-85%)	meat and fish (0-80%)	rice (0-70%)		
Hess et al. 2015 (16)	UK	average	milk (18%)	rice (12%)				
Marrin 2016 (30)	USA	average	plant based foods (55%)					
Mekonnen and Hoekstra 2012 (35)	USA	average					meat (37%)	
Song et al. 2015 (43)	China	average					pork meat (22%)	rice (22%)
Thaler et al. 2014 (48)	Austria	average	plant based foods (75%)		animal source foods (83%)			
Vanham et al 2016 (58)	Mediterranean (8 countries)	average					meat	
Vanham et al. 2013 (52)	EU (28 countries)	average	milk (exc butter) (13%)	pigmeat (12%)	milk (exc butter) (13%)	bovine meat (12%)		
Vanham et al. 2013 (61)	EU (28 countries)	average					meat	milk and milk products
Vanham and Bidoglio 2014 (55)	Italy	average					meat	crop oils
Vanham et al. 2015 (57)	EU (28 countries)	average	meat (30%)	sugar (11%)	meat (37%)	cereals (10%)		
Vanham et al. 2016 (62)	The Netherlands	average					meat (29-31%)	milk and milk products
Vanham et al. 2016 (58)	China	average	tree nuts (25%)	freshwater fish (11%)	meat	cereals		
Vanham et al. 2017(60)	Nordic region (5 countries)	average					meat (32%)	milk and milk products (19%)
Vanham 2013 (50)	Austria	average					meat	milk and milk products
Yoo et al. 2016 (63)	South Korea	average	cereals (65-75%)	tree nuts, oil crops and sugars (9-15%)	meats (35-42%)	oils and fats (18-25%)		
Yuan et al. 2016 (67)	China	average					animal source foods	

Zhuo et al. 2016 (68)	China	average					animal products (44%)	cereals (32%)
Birney et al. 2017 (1)	USA	healthy	fruit (27%)	meat, poultry, eggs (16%)	meat, poultry, eggs (39%)	dairy (27%)		
Blas et al. 2016 (3)	USA, Spain	healthy	olive oil (24-29%)	soy milk (21%)	oilive oil (22%)	beef meat (7-19%)		
Saez-Almendros et al. 2013 (40)	Spain	healthy					vegetables (34%)	cereals (17%)
Vanham et al. 2013 (52)	EU (28 countries)	healthy	meat	fruit				
Vanham et al. 2013(61)	EU (28 countries)	healthy					meat	milk and milk products
Vanham and Bidoglio 2014 (55)	Italy	healthy					meat	cereals
Vanham et al. 2016 (58)	The Netherlands	healthy					stimulants	milk and milk products
Vanham et al.2017 (59)	China	healthy	tree nuts	cereals	cereals	meat		
Vanham et al. 2017 (60)	Nordic region (5 countries)	healthy					meat (31%)	stimulants (32%)
Vanham 2013 (50)	Austria	healthy					meat	milk and milk products
Goldstein et al. (11)	USA	reduced ASF	frutis and vegetables (18%)	proteins (21%)				
Vanham et al. 2013 (52)	EU (28 countries)	reduced ASF	milk and milk products	fruit				
Vanham et al. 2013 (61)	EU (28 countries)	reduced ASF					milk and milk products	stimulants
Vanham and Bidoglio 2014 (55)	Italy	reduced ASF					cereals	crop oils
Vanham et al. 2016 (58)	The Netherlands	reduced ASF					stimulants	milk and milk products
Vanham et al. 2017 (59)	China	reduced ASF	tree nuts	cereals	cereals	fruit		
Vanham et al. 2017 (60)	Nordic region (5 countries)	reduced ASF					stimulants (29-31%0	pulses, nuts and oilcrops (14-24%
Vanham 2013 (50)	Austria	reduced ASF					milk and milk products	cereals
Goldstein et al. 2017 (11)	USA	no ASF	fruits and vegetables (34%)	grains (25%)				

* Top two items based on food groups reported in the study. If available percentages are reported. For studies that estimated multiple dietary water footprints, ranges in percentage contribution are presented. If percentage contributions could not be calculated (e.g. because data was displayed graphically), food groups are listed; only food groups that are clear major contributors across all diets are presented

Supplemental Table 7 Results from the meta-analysis on the effect of diet pattern on dietary total water footprint

	Model	Diet pattern	Coefficient (log)	*P* value	Lower 95% Confidence Limit (log)	Upper 95% Confidence Limit (log)	Coefficient (after exponentiation)	Lower 95% Confidence Limit (after exponentiation)	Upper 95% Confidence Limit (after exponentiation)	*N* estimates	*N* studies
all studies	simple	no animal source foods	-0.2886818	<0.001	-0.3361521	-0.2412115	-25.0749421	-28.54855801	-21.43245615	1933	32
		reduced animal source foods	-0.1952873	<0.001	-0.2259367	-0.1646379	-17.74017084	-20.22314012	-15.1799218	1933	32
		healthy	-0.0612204	<0.001	-0.0954	-0.0270409	-5.938409482	-9.099074197	-2.667856814	1933	32
	adjusted for location	no animal source foods	-0.2896898	<0.001	-0.3169606	-0.262419	-25.15042851	-27.16405481	-23.08113339	1933	32
		reduced animal source foods	-0.1959683	<0.001	-0.2135844	-0.1783522	-17.79617071	-19.23160113	-16.33522957	1933	32
		healthy	-0.061654	<0.001	-0.0813029	-0.0420051	-5.979185746	-7.808559872	-4.113510965	1933	32
	fully adjusted	no animal source foods	-0.2900833	<0.001	-0.3173443	-0.2628223	-25.17987602	-27.1919966	-23.11214851	1933	32
		reduced animal source foods	-0.1963015	<0.001	-0.2139077	-0.1786952	-17.82355647	-19.25770933	-16.36392167	1933	32
		healthy	-0.0622541	<0.001	-0.0818882	-0.0426201	-6.035590711	-7.862503734	-4.172463026	1933	32
excluding studies with >500 estimates	simple	no animal source foods	0.3701012	0.193	-0.1873707	0.927573	44.78811328	-17.08636814	152.8365382	337	30
		reduced animal source foods	-0.4252939	<0.001	-0.4867404	-0.3638475	-34.64223296	-38.53734307	-30.50028263	337	30
		healthy	-0.1940069	<0.001	-0.2588426	-0.1291712	-17.6347779	-22.80554825	-12.11765019	337	30
	adjusted for location	no animal source foods	0.3194119	0.151	-0.1162489	0.7550727	37.63181136	-10.97463884	112.7766206	337	30
		reduced animal source foods	-0.4287764	<0.001	-0.4728375	-0.3847153	-34.86944552	-37.6768662	-31.93556119	337	30

		healthy	-0.1964706	<0.001	-0.2429688	-0.1499725	-17.83745133	-21.57040166	-13.92683538	337	30
	fully adjusted	no animal source foods	0.3253053	0.157	-0.1253653	0.7759758	38.4453255	-11.78254147	117.2711225	337	30
		reduced animal source foods	-0.4301385	<0.001	-0.4740442	-0.3862327	-34.95809945	-37.75202616	-32.03876385	337	30
		healthy	-0.198678	<0.001	-0.2449476	-0.1524084	-18.01861691	-21.7254447	-14.13624584	337	30
excluding studies of low quality	simple	no animal source foods	-0.2884263	<0.001	-0.3357722	-0.2410804	-25.0557963	-28.52140845	-21.42215527	1918	27
		reduced animal source foods	-0.1957239	<0.001	-0.2263218	-0.1651259	-17.77607764	-20.25385627	-15.2213039	1918	27
		healthy	-0.0616445	<0.001	-0.0957599	-0.0275291	-5.978292544	-9.131783553	-2.715362769	1918	27
	adjusted for location	no animal source foods	-0.2904166	<0.001	-0.3172949	-0.2635383	-25.20480941	-27.18839979	-23.16718051	1918	27
		reduced animal source foods	-0.1965849	<0.001	-0.2139658	-0.1792039	-17.84684197	-19.26240032	-16.40645652	1918	27
		healthy	-0.062207	<0.001	-0.0815944	-0.0428195	-6.031164883	-7.835429761	-4.191569132	1918	27
	fully adjusted	no animal source foods	-0.2914613	<0.001	-0.318352	-0.2645705	-25.28290714	-27.26532827	-23.24644643	1918	27
		reduced animal source foods	-0.1970267	<0.001	-0.2144106	-0.1796428	-17.88312922	-19.29830442	-16.44313768	1918	27
		healthy	-0.0626953	<0.001	-0.0820832	-0.0433075	-6.077038664	-7.880468794	-4.23831224	1918	27

Supplemental Table 8 Results from the meta-analysis on the effect of diet pattern on dietary green water footprint

	Model	Diet pattern	Coefficient (log)	*P* value	Lower 95% Confidence Limit (log)	Upper 95% Confidence Limit (log)	Coefficient (after exponentiation)	Lower 95% Confidence Limit (after exponentiation)	Upper 95% Confidence Limit(after exponentiation)	*N* estimates	*N* studies
all studies	simple	no animal source foods	-0.300076	<0.001	-0.3525648	-0.2475873	-25.92380794	-29.71169785	-21.93179357	1834	20
		reduced animal source foods	-0.1977656	<0.001	-0.2322	-0.1633312	-17.94378296	-20.72124501	-15.06901496	1834	20
		healthy	-0.0591573	0.002	-0.0974991	-0.0208155	-5.744150696	-9.289684205	-2.060035286	1834	20
	adjusted for location	no animal source foods	-0.302352	<0.001	-0.3295288	-0.2751753	-26.09221363	-28.07374299	-24.05610181	1834	20
		reduced animal source foods	-0.199059	<0.001	-0.2168913	-0.1812267	-18.04984587	-19.49825301	-16.57537864	1834	20
		healthy	-0.0601711	<0.001	-0.0800315	-0.0403107	-5.839658854	-7.691273132	-3.950903176	1834	20
	fully adjusted	no animal source foods	-0.3030494	<0.001	-0.3302222	-0.2758767	-26.14373895	-28.12359937	-24.10935018	1834	20
		reduced animal source foods	-0.1993074	<0.001	-0.2171266	-0.1814882	-18.07019976	-19.51719284	-16.59719132	1834	20
		healthy	-0.0604242	<0.001	-0.0802695	-0.0405789	-5.863487821	-7.713239995	-3.976660089	1834	20
excluding studies with >500 estimates	simple	no animal source foods	0.2950423	0.512	-0.5877966	1.177881	34.31831741	-44.44499639	224.7485485	238	18
		reduced animal source foods	-0.431837	<0.001	-0.4977598	-0.3659141	-35.06847936	-39.21090672	-30.64376243	238	18
		healthy	-0.1940312	<0.001	-0.2617137	-0.1263487	-17.63677935	-23.02686337	-11.86925187	238	18
	adjusted for location	no animal source foods	-0.1812176	0.146	-0.4252626	0.0628273	-16.57461947	-34.64018723	6.484292498	238	18
		reduced animal source foods	-0.4364895	<0.001	-0.4727384	-0.4002405	-35.3698716	-37.67068967	-32.98411466	238	18
		healthy	-0.1907013	<0.001	-0.2277151	-0.1536875	-17.36206092	-20.3648892	-14.24600396	238	18

		no animal source foods	-0.1849332	0.127	-0.4223726	0.0525062	-16.88401965	-34.45102416	5.390909629	238	18
		reduced animal source foods	-0.4413345	<0.001	-0.4767762	-0.4058928	-35.68224723	-37.92185554	-33.36184003	238	18
	fully adjusted	healthy	-0.197003	<0.001	-0.2333041	-0.1607018	-17.88118303	-20.80872838	-14.84540357	238	18
excluding studies of low quality	simple	no animal source foods	-0.298768	<0.001	-0.3512166	-0.2463194	-25.82685288	-29.61687125	-21.83274812	1828	18
		reduced animal source foods	-0.1975808	<0.001	-0.2319779	-0.1631836	-17.92861757	-20.70363524	-15.05647822	1828	18
		healthy	-0.0579475	0.003	-0.096276	-0.0196191	-5.630050964	-9.17866854	-1.94278979	1828	18
	adjusted for location	no animal source foods	-0.3022482	<0.001	-0.3294116	-0.2750848	-26.0845416	-28.06531274	-24.04922858	1828	18
		reduced animal source foods	-0.1991499	<0.001	-0.2169821	-0.1813176	-18.0572948	-19.50556223	-16.58296159	1828	18
		healthy	-0.05999	<0.001	-0.0798699	-0.0401101	-5.822604872	-7.676354836	-3.931633794	1828	18
	fully adjusted	no animal source foods	-0.304015	<0.001	-0.3312219	-0.2768082	-26.21502014	-28.1954183	-24.18000941	1828	18
		reduced animal source foods	-0.2002667	<0.001	-0.2181066	-0.1824268	-18.14875733	-19.59602735	-16.67543647	1828	18
		healthy	-0.0611185	<0.001	-0.0810019	-0.041235	-5.928824117	-7.780806072	-4.03964034	1828	18

Supplemental Table 9 Results from the meta-analysis on the effect of diet pattern on dietary blue water footprint

	Model	Diet pattern	Coefficient (log)	*P* value	Lower 95% Confidence Limit (log)	Upper 95% Confidence Limit (log)	Coefficient (after exponentiation)	Lower 95% Confidence Limit (after exponentiation)	Upper 95% Confidence Limit(after exponentiation)	*N* estimates	*N* studies
all studies	simple	no animal source foods	-0.1093409	0.056	-0.2216032	0.0029215	-10.35752258	-19.87667694	0.292577174	1865	24
		reduced animal source foods	-0.051027	0.175	-0.1247483	0.0226942	-4.974698665	-11.7280945	2.295367248	1865	24
		healthy	0.0147133	0.725	-0.0672679	0.0966945	1.482207342	-6.50553039	10.15238067	1865	24
	adjusted for location	no animal source foods	-0.1219144	<0.001	-0.1556638	-0.0881651	-11.47758596	-14.41531223	-8.439030292	1865	24
		reduced animal source foods	-0.0568063	<0.001	-0.0789815	-0.0346311	-5.522294504	-7.594298066	-3.403830619	1865	24
		healthy	0.0057747	0.647	-0.0189472	0.0304965	0.579140572	-1.876883012	3.096628166	1865	24
	fully adjusted	no animal source foods	-0.123339	<0.001	-0.1570884	-0.0895896	-11.6036052	-14.53714937	-8.569366039	1865	24
		reduced animal source foods	-0.0570722	<0.001	-0.0792389	-0.0349055	-5.547412786	-7.618080233	-3.430332972	1865	24
		healthy	0.0057142	0.65	-0.0189944	0.0304228	0.573055718	-1.881514314	3.089030225	1865	24
excluding studies with >500 estimates	simple	no animal source foods	0.8787103	0.079	-0.100145	1.857566	140.7792373	-9.529377388	540.8120409	269	22
		reduced animal source foods	-0.2133064	0.027	-0.4025342	-0.0240786	-19.20914439	-33.13765284	-2.379102328	269	22
		healthy	-0.0430928	0.661	-0.2356312	0.1494456	-4.217749999	-20.99280012	16.11903003	269	22

	adjusted for location	no animal source foods	0.2107932	0.153	-0.0781573	0.4997438	23.46570016	-7.518105892	64.82989224	269	22
		reduced animal source foods	-0.242439	<0.001	-0.288389	-0.196489	-21.52883865	-25.05300083	-17.8389631	269	22
		healthy	-0.0913167	<0.001	-0.1388692	-0.0437643	-8.727139603	-12.96581383	-4.28204619	269	22
	fully adjusted	no animal source foods	0.1323361	0.396	-0.1733928	0.4380651	14.14919102	-15.91927191	54.970579	269	22
		reduced animal source foods	-0.2409706	<0.001	-0.2870562	-0.194885	-21.41352695	-24.95304487	-17.70707105	269	22
		healthy	-0.0872722	<0.001	-0.1348308	-0.0397136	-8.357238992	-12.61362431	-3.893535134	269	22
excluding studies of low quality	simple	no animal source foods	-0.1087077	0.058	-0.2211224	0.0037071	-10.30074299	-19.83814438	0.371397979	1859	22
		reduced animal source foods	-0.0505226	0.18	-0.1243716	0.0233264	-4.926755812	-11.69483621	2.360058826	1859	22
		healthy	0.0168003	0.689	-0.0654221	0.0990227	1.694221868	-6.332798933	10.40913621	1859	22
	adjusted for location	no animal source foods	-0.1217487	<0.001	-0.1555261	-0.0879713	-11.46291658	-14.4035264	-8.421284056	1859	22
		reduced animal source foods	-0.0566116	<0.001	-0.0788115	-0.0344116	-5.503897904	-7.578587761	-3.382625433	1859	22
		healthy	0.0062309	0.622	-0.0185462	0.0310079	0.625035244	-1.837527752	3.149365265	1859	22
	fully adjusted	no animal source foods	-0.1231555	<0.001	-0.1569442	-0.0893668	-11.58738297	-14.52482474	-8.548993025	1859	22
		reduced animal source foods	-0.0568623	<0.001	-0.0790602	-0.0346643	-5.527585107	-7.601570108	-3.407037559	1859	22
		healthy	0.0062815	0.619	-0.0184878	0.0310507	0.630126999	-1.831794896	3.153780153	1859	22

Supplemental References

1. Birney CI, Franklin KF, Davidson FT, Webber ME. An assessment of individual foodprints attributed to diets and food waste in the United States. Environmental Research Letters 2017;12(10). doi: 10.1088/1748-9326/aa8494.
2. Tom MS, Fischbeck PS, Hendrickson CT. Energy use, blue water footprint, and greenhouse gas emissions for current food consumption patterns and dietary recommendations in the US. Environment Systems and Decisions 2016;36(1):92-103. doi: 10.1007/s10669-015-9577-y.
3. Blas A, Garrido A, Willaarts B. Evaluating the water footprint of the Mediterranean and American diets. Water (Switzerland) 2016;8(10). doi: 10.3390/w8100448.
4. FAO. Detailed Trade Matrix. Internet: http://www.fao.org/faostat/en/#data/TM.
5. Capone R, El-Bilali H, Debs P, Lorusso F, Berjan S. Food environmental sustainability in Bosnia, Italy and Serbia: water, ecological and carbon footprints. Third International Scientific Symposium Agrosym 2012.
6. Damerau K, Patt A, van Vliet O. Water saving potentials and possible trade-offs for future food and energy supply. Global Environmental Change-Human and Policy Dimensions 2016;39:15-25. doi: 10.1016/j.gloenvcha.2016.03.014.
7. Davis K, Gephart J, Emery KA, Leach A, Galloway J, D'Odorico P. Meeting future food demand with current agricultural resources. Global Environmental Change 2016;39:125-32. doi: 10.1016/j.gloenvcha.2016.05.004.
8. Djanibekov N, Frohberg K, Djanibekov U. Income-based projections of water footprint of food consumption in Uzbekistan. Global and Planetary Change 2013;110:130-42. doi: 10.1016/j.gloplacha.2013.08.015.
9. Gephart JA, Davis KF, Emery KA, Leach AM, Galloway JN, Pace ML. The environmental cost of subsistence: Optimizing diets to minimize footprints. The Science of the total environment 2016;553:120-7. doi: https://dx.doi.org/10.1016/j.scitotenv.2016.02.050.
10. Gephart JA, Pace ML, D'Odorico P. Freshwater savings from marine protein consumption. Environmental Research Letters 2014;9(1):014005.
11. Goldstein B, Moses R, Sammons N, Birkved M. Potential to curb the environmental burdens of American beef consumption using a novel plant-based beef substitute. PLoS ONE [Electronic Resource] 2017;12(12):e0189029.
12. Hadjikakou M, Chenoweth J, Miller G. Estimating the direct and indirect water use of tourism in the eastern Mediterranean. Journal of environmental management 2013;114:548-56. doi: https://dx.doi.org/10.1016/j.jenvman.2012.11.002.
13. Hai-yang S. The comparison of virtual water consumption among the various consumption patterns of diet. Advance Journal of Food Science and Technology 2015;7(11):875-80.
14. Harris F, Green R, Joy EJ, Kayatz B, Haines A, Dangour A. The water use of Indian diets and socio-demographic factors related to dietary blue water footprint. Science of the Total Environment 2017;587-588:128-36. doi: 10.1016/j.scitotenv.2017.02.085.
15. Ebrahim S, Kinra S, Bowen L, Andersen E, Ben-Shlomo Y, Lyngdoh T, Ramakrishnan L, Ahuja R, Joshi P, Das SM. The effect of rural-to-urban migration on obesity and diabetes in India: a cross-sectional study. PLoS medicine 2010;7(4):e1000268.
16. Hess T, Andersson U, Mena C, Williams A. The Impact of Healthier Dietary Scenarios on the Global Blue Water Scarcity Footprint of Food Consumption in the UK. Food Policy 2015;50(0):1-10. doi: 10.1016/j.foodpol.2014.10.013.
17. Audsley E, Brander M, Chatterton JC, Murphy-Bokern D, Webster C, Williams AG. How low can we go? An assessment of greenhouse gas emissions from the UK food system and the scope reduction by 2050. Report for the WWF and Food Climate Research Network. 2010.
18. Pfister S, Koehler A, Hellweg S. Assessing the environmental impacts of freshwater consumption in LCA. Environmental science & technology 2009;43(11):4098-104.
19. HMRC. Internet: www.uktradeinfo.com/Statistics/BuildYourOwnTables/Pages/Home.aspx (accessed 13th March 2013).
20. INTRACEN. Internet: http://www.intracen.org/exporters/ (accessed 29th August 2013).
21. Jalava M, Guillaume J, Kummu M, Porkka M, Siebert S, Varis O. Diet change and food loss reduction: What is their combined impact on global water use and scarcity? Earth's Future 2016;4(3):62-78. doi: 10.1002/2015EF000327.

22. WHO. Diet, nutrition, and the prevention of chronic diseases: report of a joint WHO/FAO expert consultation: World Health Organization, 2003.
23. Jalava M, Kummu M, Porkka M, Siebert S, Varis O. Diet change - A solution to reduce water use? Environmental Research Letters 2014;9(7). doi: 10.1088/1748-9326/9/7/074016.
24. Kang JF, Lin JY, Zhao XF, Zhao SN, Kou LM. Decomposition of the Urban Water Footprint of Food Consumption: A Case Study of Xiamen City. Sustainability 2017;9(1). doi: 10.3390/su9010135.
25. Software. F. Internet: http://www.fao.org/nr/water/infores_databases_cropwat.html (accessed 25 July 2016).
26. Hoekstra AY, Chapagain AK. Water footprints of nations: water use by people as a function of their consumption pattern. Edtion ed. Integrated assessment of water resources and global change: Springer, 2006:35-48.
27. Kummu M, de Moel H, Porkka M, Siebert S, Varis O, Ward PJ. Lost food, wasted resources: global food supply chain losses and their impacts on freshwater, cropland, and fertiliser use. The Science of the total environment 2012;438:477-89. doi: https://dx.doi.org/10.1016/j.scitotenv.2012.08.092.
28. Li J. Scenario analysis of tourism's water footprint for China's Beijing–Tianjin–Hebei region in 2020: implications for water policy. Journal of Sustainable Tourism 2017:1-19. doi: 10.1080/09669582.2017.1326926.
29. Lyakurwa FS. Quantitative modeling of the water footprint and energy content of crop and animal products consumption in Tanzania. Independent Journal of Management and Production 2014;5(2):511-26.
30. Marrin DL. Using water footprints to identify alternatives for conserving local water resources in california. Water (Switzerland) 2016;8(11). doi: 10.3390/w8110497.
31. Fulton J, Cooley H, Gleick PH. California's water footprint. Pacific Institute for Studies in Development, Environment, and Security, Oakland CA 2012.
32. Martin M, Danielsson L. Environmental implications of dynamic policies on food consumption and waste handling in the European union. Sustainability (Switzerland) 2016;8(3). doi: 10.3390/su8030282.
33. Ivanova D, Stadler K, Steen-Olsen K, Wood R, Vita G, Tukker A, Hertwich EG. Environmental impact assessment of household consumption. Journal of Industrial Ecology 2016;20(3):526-36.
34. Ecoinvent. Internet: http://ec.europa.eu/eurostat/statistics-explained/index.php/Municipal_waste_statistics2015 (accessed 13 November 2015).
35. Mekonnen M, Hoekstra A. A Global Assessment of the Water Footprint of Farm Animal Products. Ecosystems 2012;15(3):401-15. doi: 10.1007/s10021-011-9517-8.
36. Mukuve FM, Fenner RA. The influence of water, land, energy and soil-nutrient resource interactions on the food system in Uganda. Food Policy 2015;51:24-37. doi: 10.1016/j.foodpol.2014.12.001.
37. FAO WaI. The state of food insecurity in the world 2012. Economic growth is necessary but not sufficient to accelerate reduction of hunger and malnutrition. Rome: FAO, 2012.
38. Rockström J. Water for food and nature in drought–prone tropics: vapour shift in rain–fed agriculture. Philosophical Transactions of the Royal Society of London B: Biological Sciences 2003;358(1440):1997-2009.
39. Ruiter de H. Water Requirements for Food Assessed at Different Levels of Scale. 2012.
40. Saez-Almendros S, Obrador B, Bach-Faig A, Serra-Majem L. Environmental footprints of Mediterranean versus Western dietary patterns: beyond the health benefits of the Mediterranean diet. Environmental health : a global access science source 2013;12:118. doi: https://dx.doi.org/10.1186/1476-069X-12-118.
41. Garrido A, Garrido M, Berdají I, Ruiz F, De Blas C, Ruiz J, García R, Hernández C, Linares P. Indicadores de sostenibilidad de la agricultura y ganadería españolas. Fundación Cajamar, Almería Google Scholar 2012.
42. Gazulla C, Raugei M, Fullana-i-Palmer P. Taking a life cycle look at crianza wine production in Spain: where are the bottlenecks? The International Journal of Life Cycle Assessment 2010;15(4):330-7.
43. Song G, Li M, Semakula HM, Zhang S. Food consumption and waste and the embedded carbon, water and ecological footprints of households in China. Science of the Total Environment 2015;529:191-7. doi: 10.1016/j.scitotenv.2015.05.068.
44. Sun S, Wang Y, Wang F, Liu J, Luan X, Li X, Zhou T, Wu P. Alleviating Pressure on Water Resources: A new approach could be attempted. Scientific reports 2015;5:14006. doi: https://dx.doi.org/10.1038/srep14006.
45. NBSC. China Statistical Yearbook 2010. Beijing: National Bureau of Statistics of the People's Republic of China., 2011.

46. MAC. Chinese agricultural statistical Report 2010. Bejing: Ministry of Agriculture of the People's Republic of China., 2011.
47. CNS. Dietary Guidelines for Chinese Residents 2011. Lhasa: Chinese Nutrition Society, 2013.
48. Thaler S, Zessner M, Mayr MM, Haider T, Kroiss H, Rechberger H. Impacts of human nutrition on land use, nutrient balances and water consumption in Austria. Sustainability of Water Quality and Ecology 2014. doi: 10.1016/j.swaqe.2014.04.003.
49. Austria. S. Versorgungsbilanzen für tierische und pflanzliche Produkte 2001 bis 2006., 2007.
50. Vanham D. The water footprint of Austria for different diets. Water science and technology : a journal of the International Association on Water Pollution Research 2013;67(4):824-30. doi: https://dx.doi.org/10.2166/wst.2012.623.
51. Zessner M, Helmich K, Thaler S, Weigl M, Wagner K, Haider T, Mayer M, Heigl S. Nutrition and land use in Austria. Österreichische Wasser–und Abfallwirtschaft 2011;63:95-104.
52. Vanham D, Mekonnen MM, Hoekstra AY. The water footprint of the EU for different diets. Ecological Indicators 2013;32:1-8. doi: 10.1016/j.ecolind.2013.02.020.
53. Westhoek H, Rood T, van den Berg M, Janse J, Nijdam D, Reudink M, Stehfest E, Lesschen J, Oenema O, Woltjer G. The protein puzzle: the consumption and production of meat, dairy and fish in the European Union. Netherlands Environmental Assessment Agency, 2011.
54. Vanham D, Bidoglio G. The water footprint of agricultural products in European river basins. Environmental Research Letters 2014;9(6). doi: 10.1088/1748-9326/9/6/064007.
55. Vanham D, Bidoglio G. The water footprint of Milan. Water science and technology : a journal of the International Association on Water Pollution Research 2014;69(4):789-95. doi: https://dx.doi.org/10.2166/wst.2013.759.
56. Bach-Faig A, Berry EM, Lairon D, Reguant J, Trichopoulou A, Dernini S, Medina FX, Battino M, Belahsen R, Miranda G. Mediterranean diet pyramid today. Science and cultural updates. Public health nutrition 2011;14(12A):2274-84.
57. Vanham D, Bouraoui F, Leip A, Grizzetti B, Bidoglio G. Lost water and nitrogen resources due to EU consumer food waste. Environmental Research Letters 2015;10(8). doi: 10.1088/1748-9326/10/8/084008.
58. Vanham D, del Pozo S, Pekcan AG, Keinan-Boker L, Trichopoulou A, Gawlik BM. Water consumption related to different diets in Mediterranean cities. Science of the Total Environment 2016;573:96-105. doi: 10.1016/j.scitotenv.2016.08.111.
59. Vanham D, Gawlik BM, Bidoglio G. Cities as hotspots of indirect water consumption: The case study of Hong Kong. Journal of Hydrology 2017. doi: 10.1016/j.jhydrol.2017.12.004.
60. Vanham D, Gawlik BM, Bidoglio G. Food consumption and related water resources in Nordic cities. Ecological Indicators 2017;74:119-29. doi: 10.1016/j.ecolind.2016.11.019.
61. Vanham D, Hoekstra AY, Bidoglio G. Potential water saving through changes in European diets. Environment international 2013;61:45-56. doi: https://dx.doi.org/10.1016/j.envint.2013.09.011.
62. Vanham D, Mak TN, Gawlik BM. Urban food consumption and associated water resources: The example of Dutch cities. Science of the Total Environment 2016;565:232-9. doi: 10.1016/j.scitotenv.2016.04.172.
63. Yoo SH, Lee SH, Choi JY, Im JB. Estimation of potential water requirements using water footprint for the target of food self-sufficiency in South Korea. Paddy and Water Environment 2016;14(1):259-69. doi: 10.1007/s10333-015-0495-x.
64. Yoo S-H, Choi J-Y, Lee S-H, Kim T. Estimating water footprint of paddy rice in Korea. Paddy and water environment 2014;12(1):43-54.
65. Yoo S-H, Lee S-H, Choi J-Y. Estimation of Water Footprint for Upland Crop Production in Korea. Journal of the Korean Society of Agricultural Engineers 2014;56(3):65-74.
66. Lee S-H, Choi J-Y, Yoo S-H, Kim YD, Shin A. Estimation of water footprint for livestock products in Korea. Journal of the Korean Society of Agricultural Engineers 2015;57(2):85-92.
67. Yuan Q, Song GB, Zhang SS. Water Footprint of Household Food Consumption in Heilongjiang Province, China. 2016 International Conference on Sustainable Energy, Environment and Information Engineering (Seeie 2016) 2016:77-80.
68. Zhuo L, Mekonnen MM, Hoekstra AY. Water footprint and virtual water trade of China: past and future. Value of Water Research Report Series 2016;69(59).
69. Amarasinghe UA, Tushaar S, Singh OP. Changing consumption patterns: implications on food and water demand in India. (Changing consuption patterns: implications on food and water demand in India.). Research Report International Water Management Institute 2007;119.
70. Chahed J, Besbes M, Hamdane A. Virtual-water content of agricultural production and food trade balance of Tunisia. International Journal of Water Resources Development 2015;31(3):407-21. doi: 10.1080/07900627.2015.1040543.

71. Chahed J, Hamdaneb A, Besbes M. A comprehensive water balance of Tunisia: Blue water, green water and virtual water. Water International 2008;33(4):415-24. doi: 10.1080/02508060802543105.
72. Du B, Zhen L, De Groot R, Long X, Cao X, Wu R, Sun C, Wang C. Changing food consumption patterns and impact on water resources in the fragile grassland of northern China. Sustainability (Switzerland) 2015;7(5):5628-47. doi: 10.3390/su7055628.
73. Gerbens-Leenes P, Nonhebel S. Consumption patterns and their effects on land required for food. Ecological Economics 2002;42(1-2):185-99.
74. Li L, Wu X-h. Analysis and Policy on Virtual Water Condition of Agricultural Products in Heilongjiang Province [J]. Science Economy Society 2008;4.
75. Xu Zhongmin LA, Zhang Zhiqiang. Virtual Water Consumption Calculation and Analysis of Gansu Province in 2000. Acta Geographica Sinica 2003;58(6):861-9. doi: 10.11821/xb200306009.
76. Gerten D, Heinke J, Hoff H, Biemans H, Fader M, Waha K. Global water availability and requirements for future food production. Journal of Hydrometeorology 2011;12(5):885-99. doi: 10.1175/2011JHM1328.1.
77. Goldstein B, Hansen SF, Gjerris M, Laurent A, Birkved M. Ethical aspects of life cycle assessments of diets. Food Policy 2016;59:139-51. doi: 10.1016/j.foodpol.2016.01.006.
78. Fødevareinstituttet D. Danskernes kostvaner. Søborg, 2010.
79. Health. LLUSoP. Internet: http://www.vegetariannutrition.org/foodpyramid.pdf.
80. Kummu M, Gerten D, Heinke J, Konzmann M, Varis O. Climate-driven interannual variability of water scarcity in food production potential: A global analysis. Hydrology and Earth System Sciences 2014;18(2):447-61. doi: 10.5194/hess-18-447-2014.
81. Liu J, Savenije HHG. Food consumption patterns and their effect on water requirement in China. Hydrology and Earth System Sciences 2008;12(3):887-98.
82. Liu J, Zehnder AJ, Yang H. Historical trends in China's virtual water trade. Water International 2007;32(1):78-90.
83. Zimmer D, Renault D. Virtual water in food production and global trade: Review of methodological issues and preliminary results. Virtual water trade: Proceedings of the International Expert Meeting on Virtual Water Trade, Value of Water Research Report Series, 2003:1-19.
84. Marlow HJ, Harwatt H, Soret S, Sabaté J. Comparing the water, energy, pesticide and fertilizer usage for the production of foods consumed by different dietary types in California. Public Health Nutrition 2015;18(13):2425-32. doi: 10.1017/S1368980014002833.
85. Notarnicola B, Tassielli G, Renzulli PA, Castellani V, Sala S. Environmental impacts of food consumption in Europe. Journal of Cleaner Production 2017;140:753-65. doi: 10.1016/j.jclepro.2016.06.080.
86. Porkka M, Gerten D, Schaphoff S, Siebert S, Kummu M. Causes and trends of water scarcity in food production. Environmental Research Letters 2016;11(1). doi: 10.1088/1748-9326/11/1/015001.
87. Renault D, Wallender WW. Nutritional water productivity and diets. Agricultural Water Management 2000;45(3):275-96. doi: 10.1016/S0378-3774(99)00107-9.
88. Rockström J, Lannerstad M, Falkenmark M. Assessing the water challenge of a new green revolution in developing countries. Proceedings of the National Academy of Sciences of the United States of America 2007;104(15):6253-60. doi: 10.1073/pnas.0605739104.
89. Singh AK, Singh JP, Reddy KK, Muralidhar W, Singh AP. Efficient irrigation water use for food security: a case study in Mahi command area. Indian Journal of Dryland Agricultural Research and Development 2007;22(2):147-52.
